# Cation-Mediated Pseudocapacitance Dominates the Interfacial Charging of α‑Fe_2_O_3_(0001) in an Alkaline Electrolyte

**DOI:** 10.1021/acs.jpcc.5c00649

**Published:** 2025-05-30

**Authors:** Jordy J.J. Eggebeen, Marc T.M. Koper

**Affiliations:** Leiden Institute of Chemistry, 4496 Leiden University, PO Box 9502, 2300 RA Leiden, The Netherlands

## Abstract

The electric double-layer at the electrode–electrolyte interface is crucial for electrocatalytic reactions in electrochemical applications, such as water splitting. On metal oxide surfaces in aqueous electrolytes, such as α-Fe_2_O_3_(0001), proton exchange between interfacial water and surface groups (e.g., Fe–O­(H)) varies with pH and potential. This process induces pseudocapacitive charging alongside standard double-layer charging. Using impedance spectroscopy, the effect of cation concentration and pH on the adsorption pseudocapacitance originating from deprotonation of Fe–O­(H) was studied. Results show that both the double-layer capacitance and adsorption pseudocapacitance remain largely unaffected by the electrolyte concentration and pH within the ‘double-layer’ window. However, the charge transfer resistance (*R*_ct_) was found to be inversely proportional to the NaOH concentration but remained constant between pH 12 and 14 at a fixed Na^+^ concentration. The concentration-independent double-layer capacitance suggests a Helmholtz or compact-type layer, with negligible diffuse layer contributions to the capacitance. Consequently, no diffuse layer effects are expected on the reaction kinetics, whether pseudocapacitive or Faradaic. Interestingly, the correlation between cation concentration and *R*_ct_ implies that cations mediate the proton-coupled electron transfer (PCET) acid–base reactions. This results in a cation-coupled PCET (CCPCET) mechanism that determines the current in the ‘double-layer’ window. Thus, the observed current in the ‘double-layer’ window of α-Fe_2_O_3_(0001) is predominantly cation-mediated and pseudocapacitive rather than attributable to traditional double-layer charging.

## Graphical abstract

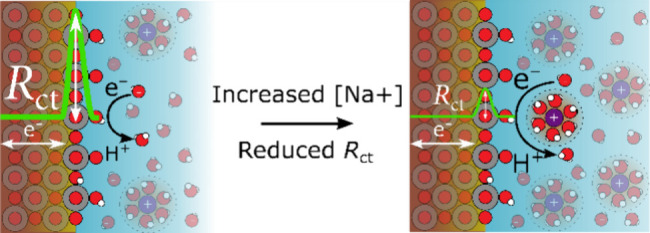

## Introduction

The interaction between an electrolyte and a (metal-)­oxide electrode is central to many electrocatalytic processes for the energy transition, such as the hydrogen evolution reaction, oxygen evolution reaction (OER) and CO_2_ reduction reaction. In the electrolyte, the electric double-layer (EDL) structure governs the local pH, active site accessibility, electron transfer kinetics, and local water structure. Classic models, such as the Gouy–Chapman-Stern model,^1–3^ describe the formation of an electrolyte structure consisting of both compact and diffuse layers. However, even with the extensions from Frumkin,^4^ Grahame,^5^ or Mott and Schottky,^6,7^ the acid–base properties and the surface charge distribution of metal oxides in the inner layer are not considered. Consequently, understanding the origin of the surface charge on an oxide electrode remains challenging due to the intricate relationship between the p*K*_a_ of the surface oxide groups and the applied potential. To obtain more insight into the fundamental properties of the oxide-electrolyte interface, well-defined single-crystalline oxides in the absence of Faradaic reactions and specifically adsorbing anions must be studied. However, defect-free and undoped single-crystalline oxides can be challenging to prepare and are difficult to maintain *in operando*. Unlike the bulk single-crystal metal electrodes often used in hanging meniscus,^8,9^ thin films with an oriented facet are typically used for metal oxide electrodes.^10–13^

Hematite (α-Fe_2_O_3_) is a versatile and readily available metal oxide that is used in many applications, such as organic pollutant degradation, photoanodes (because of its favorable bandgap of 2.2 eV), and water splitting catalysts.^14–16^ However, due to their electronic insulating nature, pure iron oxides, such as magnetite (Fe_3_O_4_), hematite or wüstite (FeO), are not readily employed for electrocatalysis.^17^ In contrast, Fe-doped materials, such as NiFeOOH and CoFeOOH, are commonly used due to the increased activity and increased conductivity.^18–22^ Despite the electronic insulating nature of pure α-Fe_2_O_3_, single crystalline α-Fe_2_O_3_ is a suitable material for investigating the electrochemical metal oxide–electrolyte interface due to its well-ordered surface (unlike many other oxides), wide accessibility, semiconducting properties, near-neutral isoelectric point and relative stability in nonacidic solutions.^23^ In particular, the α-Fe_2_O_3_(0001) surface has been studied, both experimentally and theoretically, to study the interaction of water with the metal oxide surface.^24–27^ The conditions under which α-Fe_2_O_3_ is stable *in situ* are limited, as it has been shown that α-Fe_2_O_3_(0001) irreversibly reduces to FeO_1–*x*_(111)/Fe_3_O_4_(111) under reducing conditions (700 °C in UHV) and can only be partially reoxidized by oxygen annealing at sufficient temperatures.^28,29^ Similarly, electrochemical reduction might lead to an irreversible loss of the Fe_2_O_3_(0001) surface. In water, surface oxygen defects are readily oxidized by water or OH^–^ leading to a (hydr)­oxide covered surface.^25,26^

Literature provides limited information on the typical cyclic voltammograms and electrochemical stability of single-crystalline iron oxide surfaces.^30,31^ The Pourbaix diagram, however, predicts a large stable potential window in mildly acidic to alkaline conditions at potentials above 0.4 V vs RHE.^32^ In this potential window, it is expected that the cyclic voltammetry is (partially) governed by interfacial and space-charge (SC) charging and might therefore serve well to study the properties of the Fe_2_O_3_(0001)-electrolyte interface. However, iron oxides in aqueous electrolytes undergo multiple complex multistage redox reactions which form different surface (hydr)­oxide groups.^26^ The variety of these redox reactions should be limited on single crystalline surfaces due to the presence of only specific sites. In contact with water, the Fe_2_O_3_(0001) surface is readily hydroxylated, and the two most favorable surface structures are the hydroxylated forms of the O and Fe terminations.^25,33^ As these O groups readily exchange protons with the electrolyte,^34^ pseudocapacitive charging should be expected in the whole potential range. This (pseudocapacitive) surface redox chemistry is superimposed onto the classical interfacial charging.

Without an externally applied potential, several studies have suggested that the Fe_2_O_3_(0001) surface is protonated (positively charged) below pH 4 and charge-neutral or negatively charged in pH 4–14 due to the existence of mostly μ_2_–OH^0^ which are ‘resilient’ to deprotonation.^27,35–38^ Respective p*K*_a_ values of Fe_2_O_3_(0001) have been found to be ∼19 for μ_2_–OH groups, and 8–10, −1.32 and 8 for respectively mono, double, and triple bound oxygens (μ_1_–OH_2_^+0.5^, μ_2_–OH_2_^+^ and μ_3_–OH^+0.5^). It is thus expected that μ_2_–OH^0^ is the main protonated state of the (0001) surface which is predominantly covered with μ_2_−O species.^27,39^ However, even single crystals could have step sites and defects which can affect the average surface properties. Mostly the singly coordinated hydroxide (μ_1_–OH^–0.5^) can be found around iron defects and on step sites, and they are prone to further protonation.^33^ As a result, the pH of zero charge (pH_pzc_ = 1/2 (p*K*_a,1_ + p*K*_a,2_)) is expected to lie between 8.0–9.5 for Fe_2_O_3_(0001) and might vary slightly depending on defect density, charging the surface positively below the pH_pzc_ and negatively above the pH_pzc_.^38^

The surface charge, as determined by (de)­protonation of the μ_2_–OH group, and electron transfer kinetics during (de)­protonation, could play a significant role in the charging of the interface. Previous investigations into the hematite-electrolyte interface by Boily et al. found that the surface charge is also dependent on the type of anion present, owing to specific adsorption.^27,30,40^ They focused on NH_4_Cl, NaHCO_3_ and NaCl electrolytes which were chosen for the supposedly strongly binding ammonium, nonspecifically binding (bi)­carbonate, and weaker binding chloride ions.

In this study, we investigated the Fe_2_O_3_(0001) interface in NaOH and NaClO_4_ containing electrolytes using electrochemical impedance spectroscopy (EIS) in the absence of a continuous Faradaic reaction such as the OER. Perchlorate anions were chosen because of their weak interaction with surfaces and their common use in fundamental electrochemistry. Using atomic force microscopy (AFM) we determined under which electrochemical conditions the Fe_2_O_3_(0001) surface remains sufficiently stable. The interfacial capacitance contributions were furthermore deconvoluted in the frequency domain and we demonstrate how they change with electrolyte and potential. By using a general EIS model containing the minimum number of elements, it is shown that both the double layer capacitance *C*_dl_ and the pseudocapacitance *C*_ad_ are not affected by the pH far above the pH_pzc_. Instead, it is shown that the cation concentration plays a significant role in the charge transfer barrier of the pseudocapacitance, which could be the result of a cation-mediated proton-coupled electron transfer that dominates the current in the ‘double-layer’ window of α-Fe_2_O_3_(0001).

## 2 Experimental Section

### 2.1 Materials

To prepare the electrolyte, high-purity solutions of NaOH (30%, Suprapur), NaClO_4_ (hydrate, 99.99% trace metal basis, Sigma-Aldrich), HClO_4_ (60% Suprapur, Sigma-Aldrich) and Ultrapure water (Milli-Q ≥ 18.2 MΩ cm) were used. Ar (99.999%, Lindegas) was purged through the cell for 15 min prior to the measurement. Glassware, plastic cells, and the electrode holder were stored in a 0.1–0.5 M H_2_SO_4_ (95–98%, ACS reagent, Sigma-Aldrich) solution containing 1 g L^–1^ KMnO_4_ (>99%, ACS reagent, Emsure). Thereafter, the glassware and cell parts were cleaned in diluted piranha solution (H_2_O_2_, 35%, Merck and H_2_SO_4_, 95–98%, ACS reagent, Sigma-Aldrich) and boiled in Milli-Q water (>18.2 MΩ cm) at least five times. The glass cell was used for pH < 12 and a plastic (FEP, Nalgene) cell was used for pH 12 and higher to prevent the dissolution of glass in base.^41^

Polished Fe_2_O_3_(0001) single crystals of natural origin (2 mm thickness, 5 mm diameter) were bought from SurfaceNet (Rheine, Germany). The crystals were rinsed with ethanol, acetone and Milli-Q water multiple times prior to and throughout its use. Because of possible naturally occurring dopants, the crystals were also measured using an in-house XPS to quantify the dopants. It was thereafter annealed at 1100 °C for 18 h in air prior to and after electrochemical (EC) measurements. The surface was characterized using an atomic force microscope (AFM, JPK NanoWizard 4) using soft tapping mode cantilevers with a spring constant of 2 N/m and resonance frequency of 70 kHz. All measurements were performed in air at ambient temperature and humidity and the data was analyzed using the JPK Data Processing software.

### 2.2 EC Cell Setup

Multiple attempts were made to electronically connect the hematite working electrode. First, gold wire was wrapped around a titanium wire which was pressed against the back of the crystal and heated until the gold melted. The molten gold did not wet the backside and therefore did not stick to the surface. Silver paint and epoxy were also used to adhere a small flat piece of stainless steel to the backside. In our hands, crystal detachment quickly occurred preventing proper experimentation. Therefore, a disk holder was made in-house consisting of a PEEK main body and a detachable top, separated by O-rings, which was fitted such that the crystal disk was pressed between the O-rings and the metal back contact (Figure S1). Contact was made to the back by ways of a spring-loaded brass contact (Pine Research Instrumentation) and silver epoxy. The single crystal disk fitted in the electrode holder was held in hanging meniscus configuration under potential control. The lowest vertex potential of 0.7 V was chosen to prevent reduction to Fe^2+^ and reconstruction of the surface.^28^

The three-electrode setup consisted of a Hydroflex reference, a gold (99.99%, Mateck) counter electrode and the working electrode. A 10 μF shunt capacitor was connected in parallel with the reference electrode to a gold wire (99.99%, Mateck) submerged in the electrolyte to circumvent EIS artifacts arising from the reference electrode.^42^

### 2.3 Electrochemical experiments

Electrochemical experiments were performed on a Biologic VSP-300. Prior to sweeping, the potential was kept at 1.1 V for >30 s to generate a steady-state surface and prevent unwanted side reactions such as Fe reduction at low uncontrolled potentials (OCP). Cyclic voltammetry (CV) was then used to sweep between 0.7 V and mild OER potentials (where *j* < 10 μA cm^–2^). Next, EIS was performed at various potentials between 0.7–1.55 V and the spectra were fitted to the equivalent circuits described in the text using the freely available python Impedance package.^43^ More information regarding the use of equivalent circuits can be found in the Supporting Information Section 1. The swept frequency range varied between 100 kHz–10 mHz with an amplitude of 10 mV, where the lowest probed frequency depended on the electrolyte concentration.

## 3 Results and Discussion

### 3.1 Characterization

First, XPS was used to measure contaminations and dopants in the naturally sourced Fe_2_O_3_(0001). From the XPS survey in Figure S2, no elements except for C, Ti, and Ca were detected outside the detection limit. The contamination from C and Ca are not expected to affect the measurement, but Ti-doping is known to slightly reduce resistivity and improve charge transport across the interface through the introduction of donor states below the Fermi level of hematite.^44^ In order to obtain the best surface, a pristine crystal was used for the electrochemical measurements. The crystal was first annealed at 1100 °C for 18 h in air and characterized using the AFM. As can be seen from Figure 1a,b, a relatively flat surface was obtained after annealing three times, which contained terraces that were between 30–70 nm wide with a mean roughness variation of 0.2–0.3 nm. However, after cooling down in air, the surface still contained some small pits that were 3–5 nm deep (dark areas in Figure 1b).

**Figure 1:**
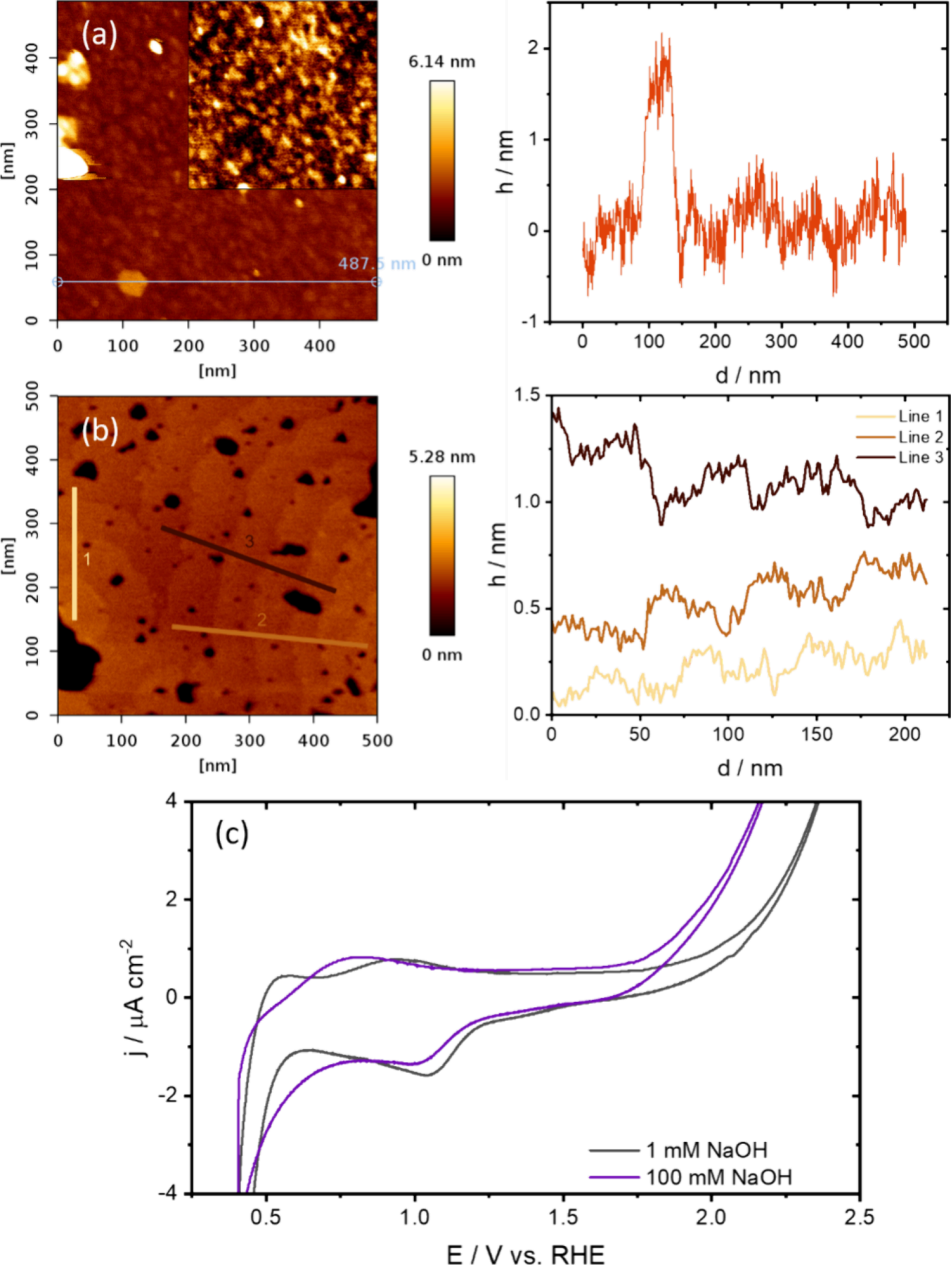
AFM images taken in air for 500 × 500 nm. (a) Pristine as-bought surface with zoom in (200 × 200 nm) and height profile. (b) Pristine surface after twice subsequent annealing at 1100 °C in air and height profile in three different areas showing the 0.2–0.3 nm steps and 30–70 wide nm terraces. (c) CV of Fe_2_O_3_(0001) in 1 and 100 mM NaOH at 50 mV s^–1^ after annealing at 1100 °C in air.

The crystal was then exposed to solutions of NaOH and scanned between 0.4–2.5 V (Figure 1c). The cyclic voltammetry response of Fe_2_O_3_(0001) shows a strong reduction current below ∼0.5 V which corresponds to the reduction of Fe^3+^ to Fe^2+^.^45,46^ Reducing iron below 0.5 V for 50 cycles in alkaline led to surface roughening, which could not be recovered completely with subsequent annealing (Figure S3).^28^

In Figure 1c, a reduction wave of Fe^3+^ to Fe^2+^ is observed below 0.5 V for both electrolytes. In 1 mM NaOH, a small oxidation peak is also present slightly above 0.5 V in the positive-going scan, which might correspond to the reverse reaction, i.e., oxidation of Fe^2+^ to Fe^3+^, because Fe^2+^ is not stable under these oxidative potentials.^23,46^ From earlier work on iron oxide films on metallic iron, it is known that Fe^3+^ starts to form at potentials as early as −0.4 V vs SCE in 1 M NaOH and might form in different iron oxide structures such as FeOOH and γ-Fe_2_O_3_.^45^ Therefore, the second anodic peak at 1.0 V in 1 mM NaOH might be Fe^2+^ to Fe^3+^ oxidation on different sites and the only anodic peak in 100 mM NaOH could also include Fe^2+^ to Fe^3+^ oxidation. Curiously, the reductive peak at 1.0–1.1 V occurs at a higher potential than the oxidation peak with seemingly no corresponding oxidation peak in both 1 and 100 mM NaOH.

AFM images were also taken after cycling, showing that the overall roughness of the surface remained roughly the same before and after cycling (Figure 2a–c versus Figure 2d–f). As can be observed in Figure 2b,e, the main changes in the surface happen on the μm scale. At this scale, somewhat dendritically shaped islands exist on the surface, which have a flat terrace-like structure on the nm scale, as illustrated in Figures 1b and 2a. Dendritic shaped islands have been observed before during oxidation of Fe_3_O_4_ to α-Fe_2_O_3_,^47^ and similar island/step edge roughness has also been observed on different iron surfaces,^48^ but, as far as we know, not on this specific surface. After cycling, the size of the dendritically shaped islands increased slightly, but the height difference (5–10 nm) between islands and the roughness on the islands remained <0.3 nm, which is most likely limited by the resolution of the AFM. While these prepared surfaces are rougher than those prepared in ultrahigh vacuum, which were not subjected to EC cycling,^28,38^ the surface roughness variation decreased significantly with annealing and remained similar after EC measurements. Due to the increase in island width, reconstruction of the surface must have taken place during cycling, e.g., through reduction to FeO, Fe_3_O_4_ and possibly γ-Fe_2_O_3_.^49^ Oxidative annealing led to a more stable and flatter surface under these conditions. The decreased roughness after cycling is in contrast to other works which showed an increase in roughness after contact with water and electrolyte due to increased hydration and electrolyte complexation.^50^

**Figure 2:**
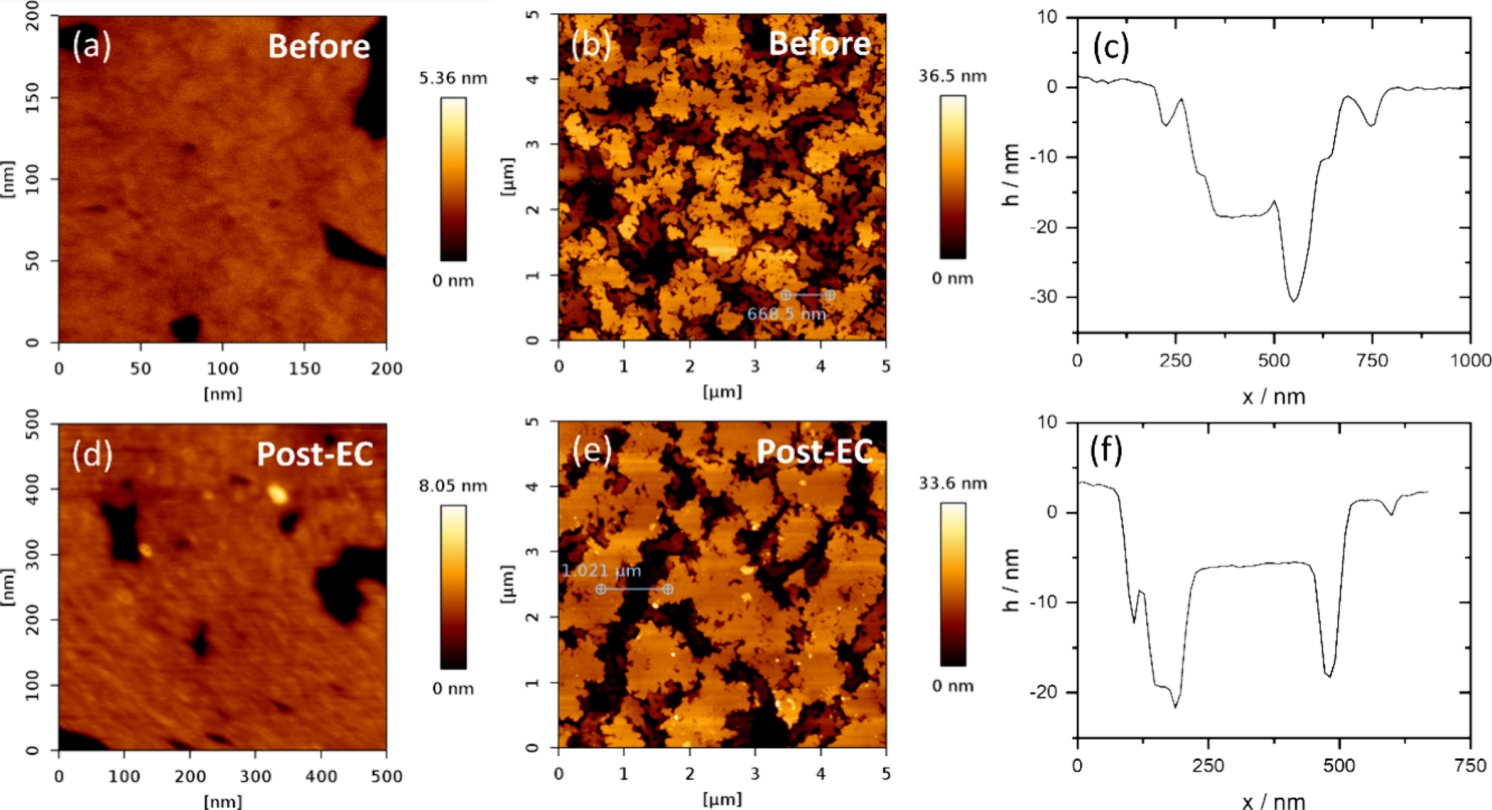
Comparison of surface roughness before and after electrochemical cycling using AFM. (a) 200 × 200 nm image of a flat terrace prior to cycling. (b) 5 × 5 μm image showing a large-scale view of the structure, revealing dendritically shaped islands. (c) Line profile corresponding to (b), illustrating the height difference between the islands and the island width. AFM images taken after electrochemical cycling: (d) 200 × 200 nm image of the flat terrace and (e) 5 × 5 μm image of the island structure. (f) Line profile associated with (e), showing the typical height difference and island widths after electrochemical cycling.

A selection of known surface transformations on both oxidized polycrystalline iron electrodes and colloidal Fe_2_O_3_/Fe_3_O_4_ have been reported in the literature.^45,46,51^ It has been shown before that Fe_2_O_3_ electrodes can be reduced into Fe_3_O_4_ or FeO/Fe­(OH)_2_ depending on the pH, redox potential and sample preparation.^46,52^ Electrochemical reduction of Fe_2_O_3_ might lead to surface defects and loss of the Fe_2_O_3_(0001) facet and therefore the potential in our experiments was kept sufficiently positive.^28^ Surface hydroxide formation on Fe_2_O_3_ is more likely to occur under mild reducing conditions as the surface becomes protonated in contact with water.^53^ Further oxidation of Fe_2_O_3_ could also lead to Fe­(IV) at sufficiently high anodic potentials, but this oxidation is not distinguishable by a clear redox feature.^18,54–56^

Figure 3a,b displays the CVs of the stable ‘double-layer’ window without any Fe^3+^ reduction between 0.7 V and the OER onset at ca. 1.7 V, at different scan rates. In this window, the current response represents a broad roughly rectangular-like capacitance window and includes a peak in the negative-going scan at ca. 0.9 V. This peak is included in the potential window because surface reduction was only observed at more negative potentials below 0.7 V. Therefore, it is assumed that the Fe oxidation state and structure do not change significantly at 0.9 V.

**Figure 3:**
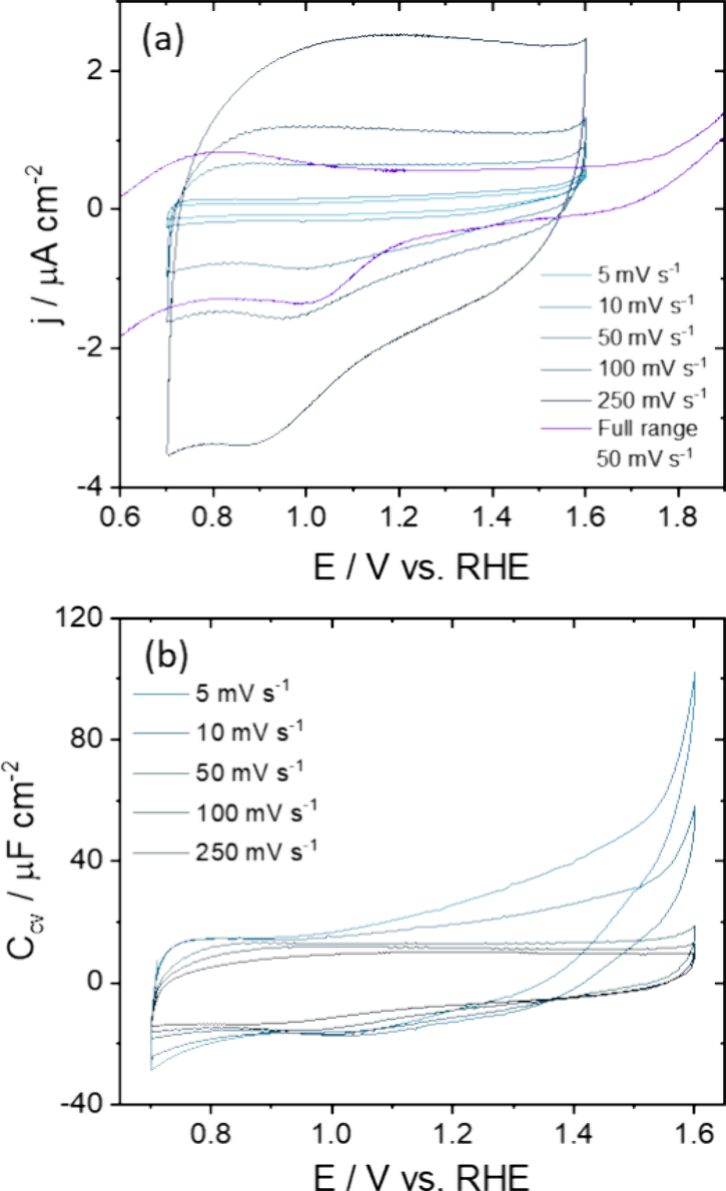
(a) Effect of scan rate on the current density. The CV from Figure 1 at 50 mV s^–1^ is also shown for comparison. (b) Voltammetric capacitance in 0.1 M NaOH, showing that the capacitance is nonlinear and scan rate dependent.

By changing the scan rate, a nonlinear current response can be observed (Figure 3a). Normalized by the scan rate, ν, the obtained voltammetric capacitance, *C*_cv_, can be obtained as follows:

$$C_{\mathrm{cv}}=\frac{j}{\nu }$$ 1

where *j* is the current density. As can be seen in Figure 3b, *C*_cv_ depends on the scan rate and is highest for the lowest scan rate, which could indicate the presence of a slow pseudocapacitive process. For ideally capacitive systems, the capacitance current scales linearly with scan rate and the interfacial capacitance is obtained as the slope of current density with scan rates. However, a significantly higher slope (10.1–12.8 μF cm^–2^) is obtained for scan rates between 5–50 mV s^–1^, compared to a slope of 6.5–9.1 μF cm^–2^ that is obtained for 50–250 mV s^–1^ (Table 1 and Figure S5).

**Table 1:** Differential Capacitance Obtained from the Slope of the Current versus Scan Rate (in Figure S5) at Three Different Potentials (0.9, 1.1, and 1.4 V) in 0.1 M NaOH

**E/V vs RHE**	**slope/μF cm**^**–2**^**(5–50** mV s^**–1**^ **)**	**slope/μF cm**^**–2**^**(50–250** mV s^**–1**^ **)**
0.9	12.8 ± 0.3	6.5 ± 0.6
1.1	11.8 ± 0.2	9.1 ± 0.8
1.4	10.1 ± 0.2	8.8 ± 0.8

### 3.2 Fe_2_O_3_(0001) Electrode Impedance

Considering the electronic structure of the electrode at the metal oxide-electrolyte interface is important as hematite is known to be a n-type semiconductor. For n-type semiconductors under depletion conditions, the surface has a lower density of charge carriers (electrons) compared to the bulk, while positive charges (holes in the valence band) accumulate at the surface.^57,58^ Thus, unlike for metals, the semiconductor space-charge layer plays an important role in the interfacial capacitance. The potential dependent space-charge capacitance, *C*_sc_, in case of a depletion layer is described by the Mott–Schottky equation:

$$\frac{1}{C_{\mathrm{SC}}^{2}}=\frac{2}{\varepsilon \varepsilon_{0}N_{D}e}\left(E_{\mathrm{sc}}-\frac{k_{B}T}{e}\right)$$ 2

where ε is the dielectric constant of Fe_2_O_3_(0001), ε_0_ is the dielectric constant of free space, *N*_D_ is the charge carrier density, *e* is the elementary charge, *E*_sc_ is the polarization of the SC layer, *k*_B_ is the Boltzmann constant, and *T* is the temperature. In the Mott–Schottky model, polarization of the space-charge layer with respect to the bulk, *E*_sc_, is given relative to the flat band potential, *E*_FB_, such that *E*_sc_ = *E* – *E*_FB_ where *E* is the applied potential relative to a reference electrode.^59^ At the flat band potential, there is no polarization of the space-charge layer relative to the bulk electrode, and the interfacial electron concentration is equal to the bulk, but the semiconductor can still be polarized relative to a reference electrode.

To include this space-charge capacitance in the interfacial charging, various equivalent electrical circuits (EECs) have been proposed in the literature (as summarized in Supporting Information in Table S2). They consider *C*_sc_ in parallel to the bulk electrode resistance, *R*_bulk_, which is in contrast to EIS performed on metals where the bulk electrode resistance is typically negligible.^8^

To identify the impedance of the isolated hematite electrode, a different Fe_2_O_3_(0001) crystal was pressed between two gold plates that were connected to the potentiostat. The EIS response was measured from 200 kHz to 1 Hz under ± 0.2 V vs OCP polarization and shows one single semicircle (Figure S6). This high frequency impedance was assigned to the intrinsic impedance of hematite using a parallel RC circuit with an approximate SC capacitance of 23 ± 5 nF cm^–2^ and a resistance of 10–25 kΩ cm^2^. Despite this crystal being a different crystal than the one measured in Figure 3, with similar dimensions and the same exposed facet, the high frequency EIS response is similar for both crystals and is therefore assumed to be related to the bulk charging mechanism of the semiconductor electrode. Moreover, a SC capacitance of 23 ± 5 nF cm^–2^ matches previous literature values, which has reported a capacitance around ∼100 μF for 11.8 nm films, decreasing down to ∼10 μF for 28.6 nm films,^60^ ∼3 μF cm^–2^ for 47 ± 6 μm thick films,^61^ and down to the nF cm^–2^ range for thicker single crystals.^30,31^ Based on this, *C*_sc_ should scale with film thickness until a size of the depletion region of a few 100 nm.^62^ However, it must be noted that impedance at high frequencies might also arise from the limitations of the electrochemical cell with a high impedance and therefore one has to be careful to interpret these data.^8,42,63,64^

### 3.3 Fe_2_O_3_(0001)-Electrolyte Interface

For the electrode–electrolyte interface, the impedance was measured at a potential between 0.75–1.35 V vs RHE in various NaOH concentrations between 0.01 and 1 M. The Nyquist and Bode representations of the raw data alongside the fitted model are presented in Figure 4a,b. At high frequencies (>1 kHz), the small semicircle belonging to the hematite bulk (R = 10 – 25 kΩ cm^–2^, *C* = 23 ± 5 nF cm^–2^) can be distinguished in the Nyquist plot from a larger impedance semicircle and low frequency (pseudo)­capacitance belonging to electrode–electrolyte interface. Despite the presence of the mid-to-low frequency impedance, the impedance in the high frequency region in Figure 4a,b is identical to the system without electrolyte present (Figure S6).

**Figure 4:**
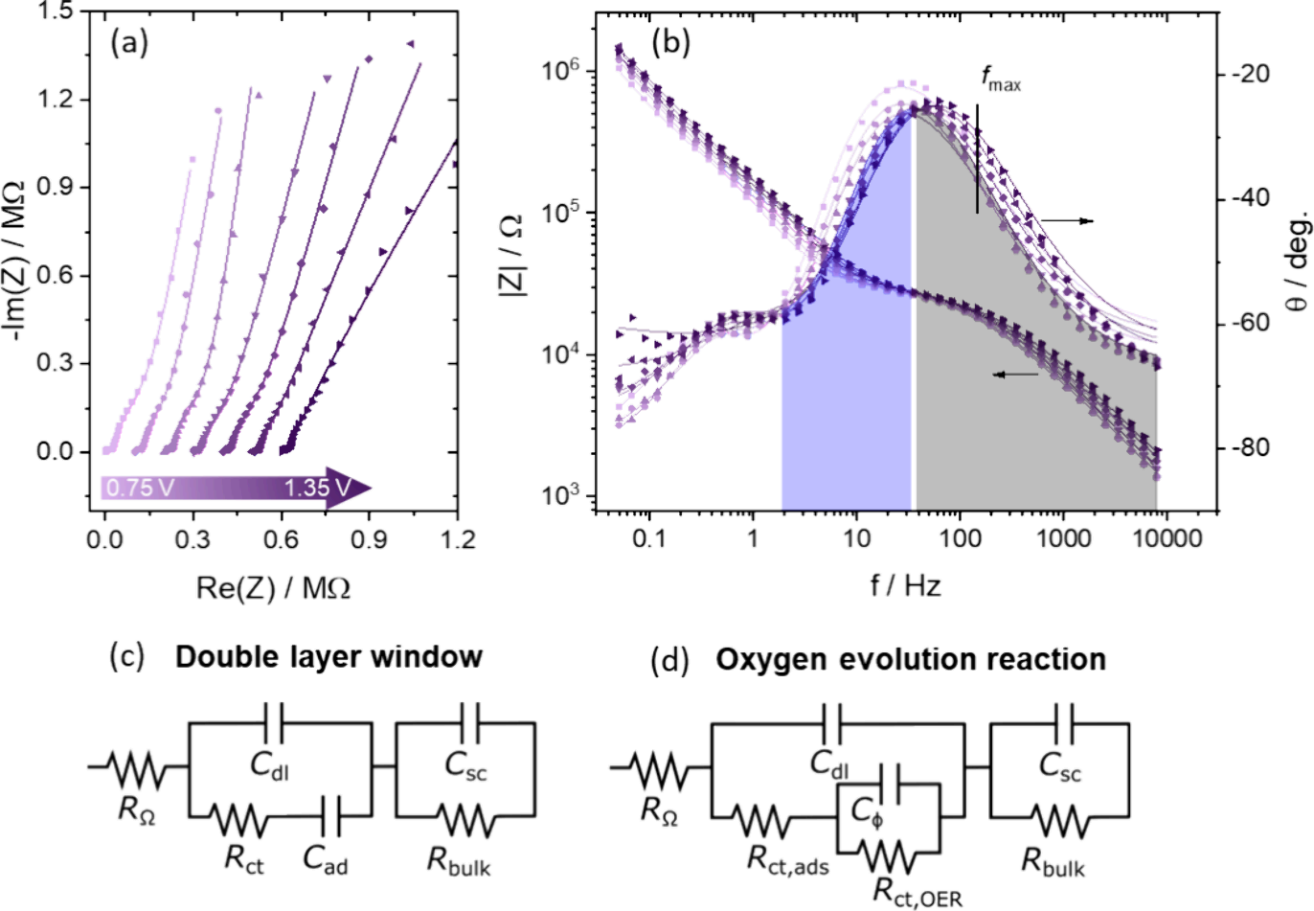
(a) Nyquist representation of the impedance data (points) and model fit (line) obtained in 0.1 M NaOH between 0.75 and 1.35 at 0.1 V spacing, with frequencies from 100 kHz to 50 mHz. Each subsequent data set is offset by 100 kΩ on the Re­(Z) axis to visualize the fit. (b) Bode plot representation of the impedance data (points) and model fit (line) obtained under the same conditions. (c) EEC used for the impedance of the α-Fe_2_O_3_/aqueous system in the ‘double-layer’ window. (d) EEC used for the impedance of a semiconductor (film) electrode with a faradaic (OER) reaction occurring, where *R*_ct,ads_ + *R*_ct.OER_ equals the total charge-transfer resistance.^65^

The EIS data was fitted using the EECs in Figure 4c,d, which model the impedance of the electrode under two different conditions. Both models include the ohmic (solution) resistance, *R*_Ω_, in series with two circuits related to the bulk electrode and electrode–electrolyte interface impedance, respectively. Both models also model the bulk electrode as a space-charge capacitance, *C*_sc_, in parallel to a bulk resistance *R*_bulk_. Figure 4c shows the EEC used to model the impedance data in the ‘double-layer’ region, where no OER current flows. In absence of any OER current, the electrode–electrolyte interface is modeled by a double layer capacitance, *C*_dl_, in parallel with a series connection of charge transfer resistance (of adsorption), *R*_ct_, and a corresponding adsorption pseudocapacitance *C*_ad_. Figure 4d shows the EEC used to model the impedance data in the OER region from ca. 1.35 V. In the OER model, the total interfacial charge transfer resistance is modeled by *R*_ct,ads_ + *R*_ct,OER_ where the former is the resistance for the adsorption pseudocapacitance, and the latter is the resistance for the OER. *R*_ct,OER_ is in parallel to an adsorption (pseudo)­capacitance, *C*_Φ_, related to the adsorption of OER intermediates.^65^ As *C*_Φ_ and *C*_ad_ refer to different adsorption processes, they are labeled distinctively. All *C*_Φ_, *C*_ad_ and *C*_sc_ were modeled with constant phase elements (CPE) to improve the fit. In contrast, treating *C*_dl_ as a CPE led to fluctuating fitting parameters, and hence it was not fitted with a CPE. For a CPE, the pre-exponential factor, *Q*, and exponential factor, α, are introduced to quantify the deviation from a perfect capacitance by defining the impedance of a CPE as

$$Z_{\mathrm{CPE}}=\frac{1}{{\left(j\omega \right)}^{\alpha }Q}$$ 3

where *j* = $\sqrt{-1}$ .^66^ The exponential factor, α*,* represents the deviation from a −90° phase angle of the impedance response such that the phase angle, θ, is −90° × α. Typical α are 0.95–1.0 for the double layer capacitance of single crystal electrodes,^8^ but are found as low as 0.6–0.8 for roughened hematite crystals,^30,67^ or even as high as 0.95 for less crystalline metal oxides.^68,69^ A more detailed analysis of the observed impedance, EEC and electronic elements can be found in the Supporting Information Section 1.

To measure the full impedance response, very low frequencies (<100 mHz) had to be probed. Therefore, a Lissajous analysis was also done to determine the linearity and stability of the EIS response, as shown in Figure S7, from which the system was concluded to be stable, down to at least 10 mHz.^70^

The Nyquist plot’s second semicircle in Figure 4a starts below ∼100 Hz as indicated by the blue-shaded area in Figure 4b. At 150 Hz, the first semicircle (gray-shaded peak in Figure 4b) reaches its maximum absolute impedance. At ∼50 Hz, the phase angle is closest to 0 which indicates the end of that semicircle and that the semiconductor properties of hematite are no longer dominating below these frequencies. At these lower frequencies, the contributions from double-layer charging at the electrode–electrolyte interface can be probed. We use *C*_ad_ for charging by adsorption by e.g. H^+^/OH^–^ and anions, and *C*_dl_ for the charging that does not involve a measurable charge transfer across the electrode–electrolyte interface. Typically, α_sc_ for the space-charge capacitance was around 0.75–0.85 but constant with potential, and α_ad_ for the adsorption pseudocapacitance was between 0.80–0.90 (see also Figure S13).

As the surface should consist of mostly μ_2_–OH sites, the most important adsorption process is likely the proton coupled electron transfer (PCET) step:

$$\mu_{2}-\mathrm{OH}↔\mu_{2}-O+H^{+}+e^{-}$$ 4

Formation of μ_2_–OH_2_ will likely not occur due to a p*K*_a_ of −1.32.^27^ Moreover, the potential is kept sufficiently positive which could prevent reduction and protonation to μ_2_–OH_2_. It is well-established that the deprotonation of surface OH groups on metal oxides is a prerequisite for water oxidation and occurs from surface states rather than holes from the valence band.^71,72^ Previous studies suggest that these surface states govern the deprotonation reaction and that the occupancy of the surface states is determined by the rate of charge transfer between electrolyte and electrode.^73,74^

Other adsorption processes can stem from the adsorption on Fe sites. Namely, both H_2_O and OH^–^ can adsorb on surface-exposed Fe sites:

$${\mathrm{Fe}}^{3+}+H_{2}O↔{\mathrm{Fe}}^{3+}-OH_{2}$$ 5

$${\mathrm{Fe}}^{3+}+{\mathrm{OH}}^{-}↔{\mathrm{Fe}}^{3+}-\mathrm{OH}+e^{-}$$ 6

where the absorbing species (H_2_O or OH^–^) depends on the electrolyte pH. How much the surface oxide is protonated will depend on the applied potential and the acid–base equilibria.^27,30,38^ Due to a very low p*K*_a_ of −1.32, Fe–OH_2_ will be deprotonated resulting in a similar Fe–OH site that would have originated from the adsorption of OH^–^.^39^ However, for the O-terminated (0001) surface, only defect Fe sites can adsorb H_2_O or OH^–^, which will lead to a very small contribution to the total adsorption pseudocapacitance.^38^ Moreover, these defect sites were observed to not significantly influence the surface potential-pH response of Fe_2_O_3_(0001).^50^

To investigate the interface sensitivity to the electrolyte, the electrolyte concentration was varied from 10 mM to 1 M NaOH, i.e., from pH 12.0 to ∼13.7. Figure 5 shows the total voltammetric capacitance as measured using cyclic voltammetry between 0.7 V and the onset of OER at ca. 1.6 V vs RHE at 10, 50, and 250 mV s^–1^.

**Figure 5:**
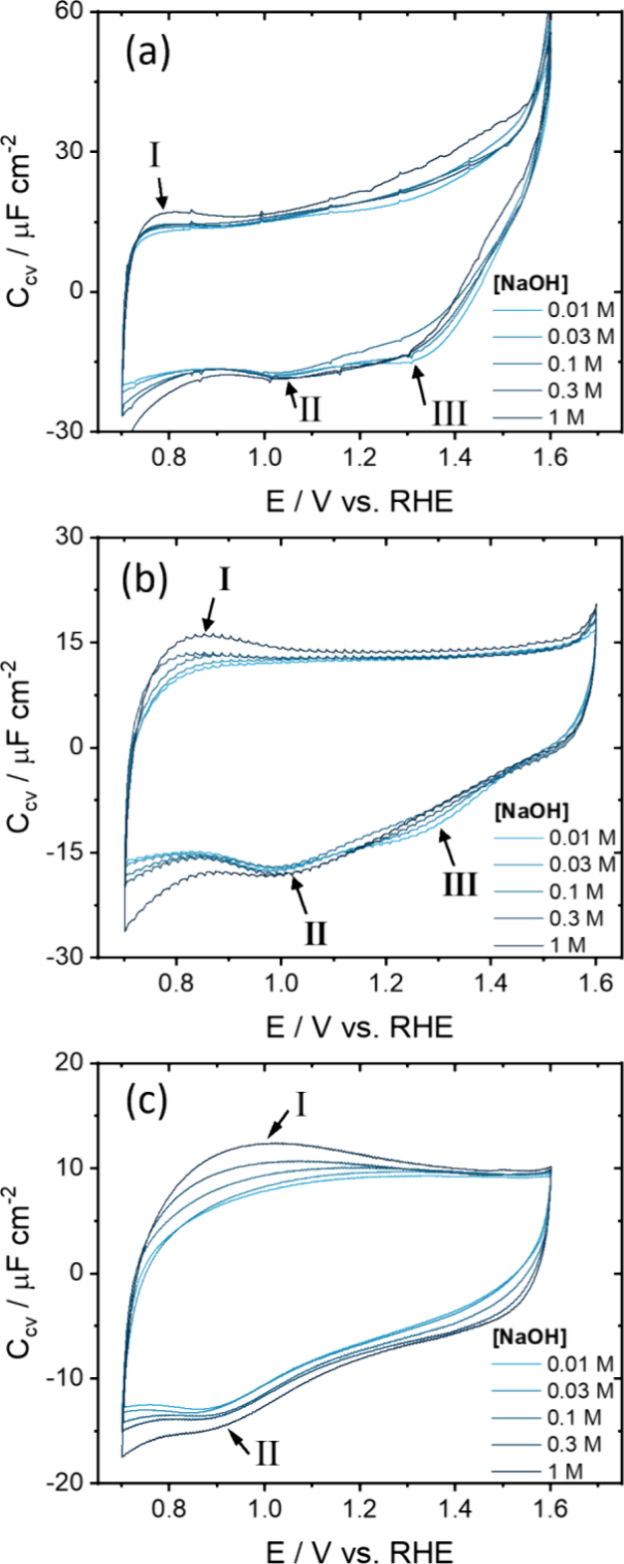
Voltammetric capacitance (obtained by normalizing the current by the scan rate) in 10–1000 mM NaOH between 0.7 and 1.6 V at a scan rate of (a) 10, (b) 50, and (c) 250 mV s^–1^.

As already illustrated in Figure 3, the voltammetric capacitance in the whole ‘double-layer’ window and the Fe redox current (peak I and reductive wave below 0.7–0.8 V) both depend on scan rate. Figure 5 shows that this is true for all pH values (NaOH concentrations) studied. For low scan rates, two reduction peaks (II and III) at 1.0 and 1.3 V are observed in Figure 5a,b, with seemingly no associated oxidation peaks. Instead of a redox couple with an oxidation and reduction peak of equal charge, the charge under peaks II and III might be related to the whole positive scan as peak II at 1.0 V becomes more prominent the higher the upper vertex potential in Figure S10a. Similarly, in Figure S10b, the positive scan capacitance increases with an increasingly lower vertex potential. However, the exact charge associated with the peak is challenging to determine due to the nonflat baseline current observed during the reverse scan.

Interestingly, peak III at 1.3 V in Figure 5b is only present for [NaOH] < 0.1 M. Peak III in Figure 5c even disappears at 250 mV s^–1^, and could be related to a significantly slower process or stem from μ_1_–OH_*x*_ defect sites with a lower p*K*_a_ than the pristine (0001) surface.^27,39^

Analysis of the peak positions revealed no apparent pH dependence on the RHE scale. Specifically, the peak positions of the reduction peaks II and III remained constant on the RHE scale, even with variations in NaOH concentration. This suggests that processes associated with these peaks demonstrate Nernstian behavior, as observed before,^38^ and there could be an underlying PCET. Similarly, the onset of the oxygen evolution reaction (OER), indicated by the current tilt above 1.55 V, remained constant on the RHE scale, reinforcing the pH independence of these processes when normalized to the RHE.

Within the ‘double-layer’ window of Fe_2_O_3_(0001), the NaOH concentration dependence varies for different processes, such as the increase in voltammetric capacitance with higher NaOH concentrations at higher scan rates in Figure 5c. Notably, currents related to the Fe redox reactions increase at increased NaOH concentrations because peak I shifts to higher potentials. The [NaOH] dependence of this Fe redox peak is more pronounced at higher scan rates (50 and 250 mV s^–1^ in Figure 5b,c) compared to 10 mV s^–1^ (Figure 5a). Other small differences in the current/capacitance around 1.2–1.5 V were observed only at 10 mV s^–1^ in Figure 5a, but these are likely attributable to noisy low currents (∼0.2 μA cm^–2^) and the highly resistive electrode used in these experiments.

Therefore, we assume that the broad (pseudo)­capacitive region between ca. 1.0 and 1.5 V likely arises from a combination of EDL charging and adsorption pseudocapacitance, potentially involving PCET reactions at μ_2_–OH sites. These reduced μ_2_–OH sites are recovered on the negative scan such that peak II is assigned to the hydrogen adsorption on μ_2_–O sites. It is possible that this hydrogen adsorption is kinetically irreversible because the width of peak II at half-maximum is larger than 90.6 mV and that hydrogen adsorption causes surface restructuring.^75^ However, the real peak height is difficult to assess and the capacitance is too low to conclude a significant change in H-coverage from an assumed full monolayer leading to a reconstruction.^76^ Moreover, in ultrahigh vacuum, oxidative surface reconstruction is only known for Fe_3_O_4_(001), whereas Fe_2_O_3_(0001) remains unreconstructed.^25,28,77^ Peak III at 1.3 V, which also remains constant on the RHE scale, might possibly be related to hydrogen adsorption on defect μ_1_-O­(H) sites present at terrace edges.^27^

Due to the convolution of EDL capacitance and adsorption pseudocapacitance, EIS was performed between 0.75–1.55 V. The full impedance spectra are shown in Figure S12. At potentials below 1.35 V, the impedance spectra could be fitted satisfactorily with the EEC in Figure 4c. At potentials above 1.35 V, i.e., the onset of OER, the EIS response is fitted with the EEC in Figure 4d, because the third time constant/semicircle cannot be modeled (see Figure S11) with the EEC in Figure 4c. The main fitting parameters are displayed in Figure 6 and the additional fitting parameters related to the bulk electrode and CPE exponents can be found in Figure S13. However, the EIS response could not be fitted accurately at 1.35 ± 0.05 V, probably due to the change in EEC around this potential, explaining the jump in data in Figure 6a–c.

**Figure 6:**
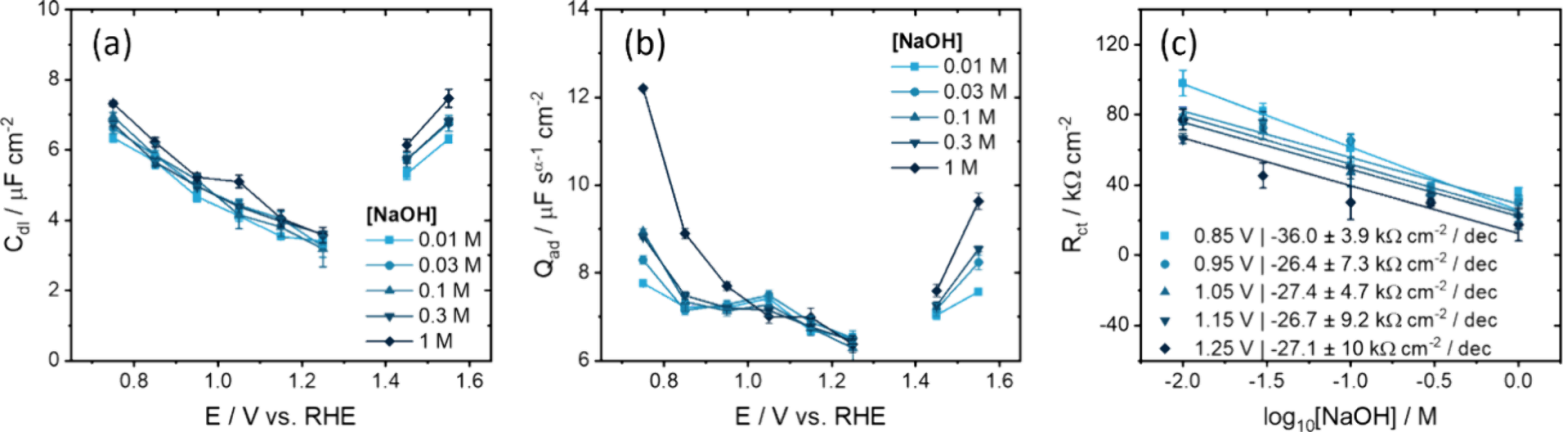
Fitting parameters of the impedance below 1.5 kHz modeled using the EECs in Figure 4c,d, showing the effect of concentration and potential on the (a) double-layer capacitance and (b) adsorption CPE pre-exponential *Q*_ad_ factor. (c) Relation between the *R*_ct_ and log_10_ [NaOH] at different potentials.

Figure 6a shows that *C*_dl_ decreases approximately linearly from ∼7 μF cm^–2^ to ∼3 μF cm^–2^ between 0.75–1.35 V, whereas it increases again above 1.45 V. Interestingly, *C*_dl_ shows a negligible NaOH concentration dependence of maximum 1 μF cm^–2^ despite the 100-fold concentration difference (Figure 6a). Moreover, *C*_dl_ is much lower than typically seen for metals,^8,9^ and *C*_dl_ is distinctly different from the voltammetric capacitance in Figure 5. Because of the negligible NaOH concentration dependence and the low capacitance value, a significant contribution of a diffuse EDL is unlikely for this system. We interpret *C*_dl_ therefore as a Helmholtz type layer.

In contrast to *C*_dl_, *Q*_ad_ is NaOH concentration dependent, but only where Fe redox (<0.85 V) and OER take place (>1.45 V), as can be seen in Figure 6b. Between 0.95 and 1.25 V, no change in pseudocapacitive behavior is observed and *Q*_ad_ only decreases by ∼0.5 μF cm^–2^ in this window. In addition to the weak potential dependence in *Q*_ad_ in this potential window, α was also found to be weakly potential dependent over the different NaOH concentrations (Figure S13). Therefore, it is likely that the actual adsorption pseudocapacitance is more strongly potential dependent, but mostly reflected by the potential-dependent α parameter. Compensation by α*,* using any of the formulas described in the Supporting Information and by previous work,^8^ would have resulted in a stronger decrease in *Q*_ad_ with potential, but this was not done due to too low α values (<0.85).

The most significant observation is the decrease in *R*_ct_ as a function of [NaOH], as shown in Figure 6c for potentials between 0.85 and 1.25 V. Outside this potential window, there is also a decrease in the apparent *R*_ct_, but it is linked to Fe redox and OER (see Figure S13d). This relation between *R*_ct_ and log_10_ [NaOH] suggests that the electron transfer rate depends on the NaOH concentration at potentials where no iron redox or OER occurs. This implies that, whereas the adsorption pseudocapacitance (*Q*_ad_) is independent of pH on the RHE scale, the reaction barrier of the corresponding PCET is significantly reduced in the presence of more OH^–^ or Na^+^. As a result of the PCET, the surface becomes more deprotonated with more positive potential gradually, rather than in one clear step or peak like seen for RuO_2_ and IrO_2_ catalysts.^10,13^ Consequently, the coverage of OH groups vs O groups changes with potential and affects the electrode–electrolyte interface.

Earlier work by Klahr et al. demonstrated that surface hydroxyl deprotonation takes place predominantly by surface trapped holes which were generated through photoexcitation.^74^ Here, no such illumination source is present and thus surface trapped holes can only be generated from thermal excitation or from band bending through the applied potential. Therefore, our charge transfer resistance (10^4^–10^5^ Ω cm^2^) is higher than those in photoelectrochemical studies (10^1^–10^3^ Ω cm^2^).^78,79^

By assuming that all the transferred charge involved in the deprotonation of μ_2_–OH is reflected by the integration of *Q*_ad_ or *C*_dl_ over the potential window,^75^ an estimation of the change in μ_2_–OH coverage over potential can be made. For the Fe_2_O_3_(0001) surface, there are roughly 13.7 O atoms nm^–2^.^35^ With one proton per electron per O-site, the total charge density of one full monolayer of protonated oxygen groups equals to about 214.8 μC cm^–2^. Based on the integration of *Q*_ad_, the average decrease in H-charge over 0.5 V in NaOH (for every concentration) was calculated to be 3.7 ± 0.2 μC cm^–2^ or 1.7 ± 0.1% of one full monolayer (ML%) (Figure S14). From integration of the CV, a charge density of 7.96 μC cm^–2^ (3.7 ML%) is obtained in 0.1 M NaOH at 10 mV s^–1^ over 0.5 V, which is slightly more than the combination of *Q*_ad_ and *C*_dl_ integrated over 0.5 V. Therefore, the pseudocapacitance is responsible for at least 47 ± 2% of the measured interfacial charging current between 0.75–1.25 V. The slight difference between the integrated voltammetric capacitance and the integrated EIS capacitances might stem from the presence of a constant phase element and an equivalent model that might not fully encompass all contributions to the current.^66^

To isolate the pH effect from the cation effect on the Fe_2_O_3_(0001)-electrolyte interface, CV and EIS were performed from pH 12 to ∼13.7 with the total Na^+^ concentration kept constant at 1 M by adding the appropriate amounts of NaClO_4_. The CVs can be found in Figure 7 and the (fitted) EIS spectra can be found in Figure S15.

**Figure 7:**
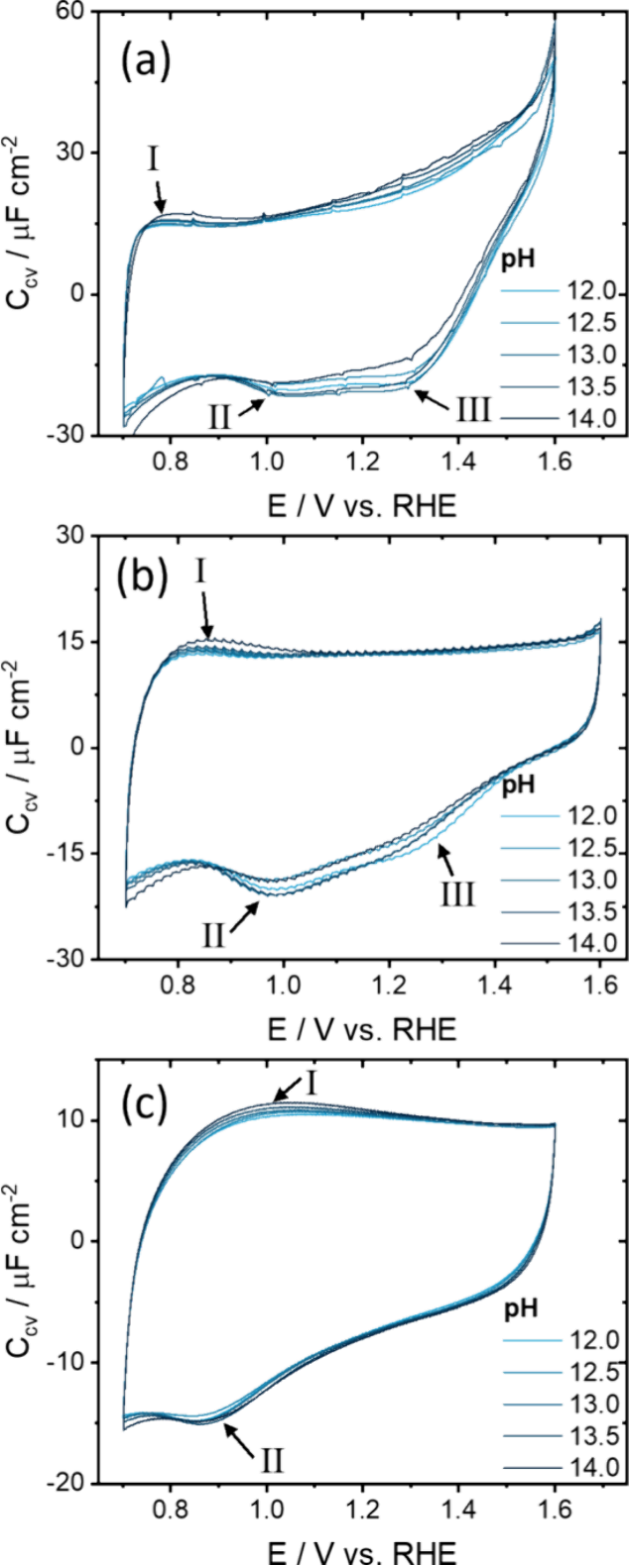
Voltammetric capacitance (obtained by normalizing the current by the scan rate) for pH 12–14 with [Na^+^] being kept constant at 1 M, measured between 0.7 and 1.6 V at a scan rate of (a) 10, (b) 50, and (c) 250 mV s^–1^.

While the overall CVs in Figure 7 look like Figure 5, differences between voltammetric capacitances for different pH values are smaller in Figure 7. Only small differences around the reduction peaks II and III in Figure 7a are observed, with the capacitance increasing slightly with pH. The forward scans of Figure 7b,c are more consistent over the whole pH range than the backward scans. Small differences in the current of the negative going scan are likely attributed to small tilts in the CV. As can be seen in Figure S16, small differences in impedance/resistance from making a meniscus as well as electrode assembly result in different contact resistances and thus tilting of the CV.

Interestingly, *C*_cv_ in Figure 7c shows no significant dependence on the pH, in contrast to Figure 5c where *C*_cv_ increased with the NaOH concentration for all potentials. As established earlier, the measured potential range consists of a simultaneous EDL and pseudocapacitive charging with an underlying electron transfer. Pseudocapacitive charging is slow due to the large barrier (*R*_ct_) and is therefore measured less at high scan rates in contrast to the double layer charging.^75,80^ Figure 5c showed that the voltammetric capacitance increased with NaOH concentration because of the reduced *R*_ct_ at higher NaOH concentrations. In other words, *R*_ct_ determines how much pseudocapacitive current can be measured at a given scan rate. As the measured capacitance does not change with pH at high scan rates when the Na^+^ concentration is fixed (Figure 7c), it can be considered that the pseudocapacitive contributions to the voltammetric capacitance are not influenced by the pH alone, but by the cation concentration as well.

This is confirmed by the EIS fitting results in Figure 8. First, *C*_dl_ and *Q*_ad_ in the ‘double-layer’ window do not change over the whole pH range (Figure 8a,b) and the α exponent remains pH-independent as well (Figure S17a). These results are in line with the apparent pH independent voltammetric capacitance in Figure 7 over the whole potential region except below 0.9 V where Fe is reduced. In contrast to Figure 6c, the plot of *R*_ct_ vs the log [OH^–^] in Figure 8c, at fixed [Na^+^], has a much smaller slope (for some potentials even zero slope). This suggests that *R*_ct_, and thus the pseudocapacitive current, is not dependent on the pH (on the RHE scale), but rather on the Na^+^ concentration.

**Figure 8:**
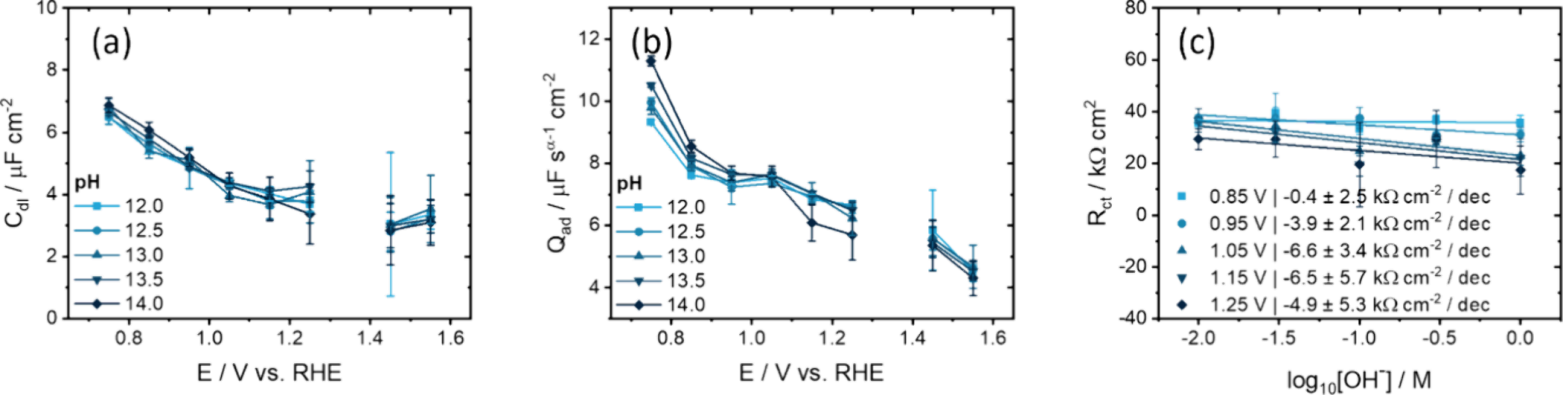
Fitting parameters of the impedance modeled using the EECs in Figure 4c,d, showing the effect of pH and potential. Potential-dependent (a) double layer capacitance and (b) adsorption CPE pre-exponential *Q*_ad_ factor and (c) plot of *R*_ct_ vs log_10_[OH^–^] that shows a negligible effect of pH on the *R*_ct_.

From these results it appears that adsorption pseudocapacitance plays a dominant role in the measured interfacial capacitance, even in a potential window of 0.8–1.25 V that one would ascribe to a ‘double-layer’ window. The observation that the capacitance and the charge-transfer resistance do not depend on pH on the RHE scale, but do depend on the cation concentration, suggest that that the PCET reaction is cation coupled. Therefore, a cation-coupled and proton-coupled electron transfer (CCPCET) step is the main contributor to the capacitive current of the Fe_2_O_3_ CV. Moreover, *C*_dl_ resembles a Helmholtz type capacitance which suggests a closely packed ion layer close to the hematite surface. The diffuse GC layer has no significant contribution to the capacitance under these conditions.

Finally, we analyzed the EIS data at high frequency to study the semiconductor charging. From the Mott–Schottky analysis (Figure S18 and Supporting Information section 2), an important role of surface states is suggested. Assuming a significant density of surface states, it would entail that surface charge is localized at the surface rather than spread out in a space-charge layer, and that the potential drop at the interface would be Helmholtz-like,^81–84^ which agrees with the above results. A more detailed analysis and discussion are given in the Supporting Information Section 2.

In summary, the electrolyte dependence of the adsorption pseudocapacitance demonstrated that cations play a significant role in the PCET acid–base reaction facilitated by the terminating μ_2_−O­(H) groups. Our Mott–Schottky plot analysis indicates the presence of surface states due to localized surface charges on terminating oxygen groups. This implies that the CCPCET is also strongly linked to the polarization of the interface, making it the dominant charging pathway and rendering space-charge-region contributions negligible. Consequently, this pathway is the primary contributor to the measured voltammetric current. Furthermore, intimate cation-surface interactions could also explain the electrolyte-independent semiconductor properties.

## 4 Conclusions

In conclusion, we have shown that the EDL at the Fe_2_O_3_(0001)-electrolyte interface in alkaline electrolyte consists of a serial semiconductor space-charge region and parallel compact Helmholtz type and adsorption pseudocapacitance layer. In this convoluted interface, the adsorption pseudocapacitance depends on the proton activity in line with Nernstian behavior and implies a proton-coupled electron transfer. We suggest that this PCET is the main dominating interfacial charging mechanism for hematite surfaces over the whole potential range in absence of other adsorbing species such as anions. Moreover, we have found that the charge transfer barrier (*R*_ct_) is not reduced at higher pH but decreases linearly with log_10_[Na^+^]. Therefore, the PCET is cation-mediated and likely involves a cation-activated OH^–^ in alkaline conditions and at a pH > pH_pzc_. In combination with the anomalously negative flat band potential that falls outside the conduction and valence band positions of hematite, it suggests that the presence of (de)­protonated μ_2_–OH sites are responsible for energy states within the bandgap. Whereas interfacial electron transfer from a semiconductor would result in band bending, these surface states enable an alternative electron conduction pathway with a reduced potential barrier. While hematite is an almost insulating semiconductor, and therefore not very practical as an electrode, it demonstrates that the interface is more conductive than thought possible due to the strong cation-surface interactions. Furthermore, this study strengthens the argument that the apparent double-layer capacitance for metal oxides is not constant and that it is not a good measure of electrochemical active surface area. Instead, we should develop a model that encompasses the whole EDL with electrode, electrolyte and adsorption as the three main charging contributors. Surface area determination should be preferably based on a surface adsorption or surface titration reaction, which are however notoriously difficult to unambiguously assign for oxide surfaces.

## Supplementary Material



## Notes

Additional CVs, AFM images, EIS analysis, fitted EIS spectra and fitting parameters, and Mott–Schottky analysis (PDF)
