# Supplementary material for: Cation-Mediated Pseudocapacitance Dominates the Interfacial Charging of α‑Fe2O3(0001) in an Alkaline Electrolyte
Source: J Phys Chem C Nanomater Interfaces. 2025 May 30;129(23):10473–85. doi: 10.1021/acs.jpcc.5c00649 (PMC12169672; doi:10.1021/acs.jpcc.5c00649)
Supplement: Supplementary file 1 [file jp5c00649_si_001.pdf]

Supplementary information to

Cation-mediated Pseudocapacitance Dominates the Interfacial Charging of α-Fe2O3(0001) in Alkaline Electrolyte

Jordy J.J. Eggebeen, Marc T.M. Koper

Leiden Institute of Chemistry, Leiden University, PO Box 9502, 2300 RA, Leiden, The Netherlands

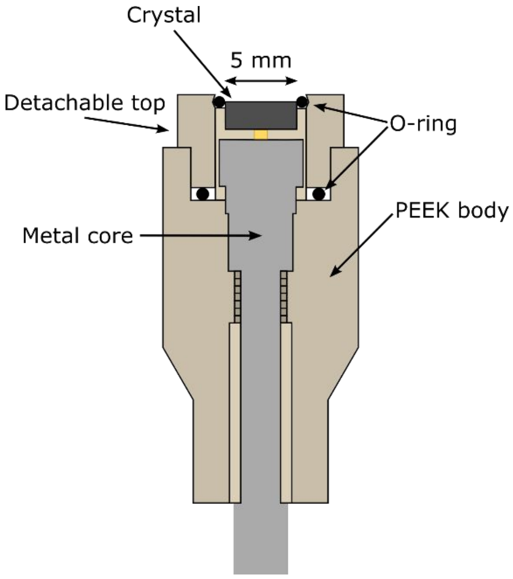

Figure S1: Schematic representation of the custom-made electrode holder in which a 5x2 mm Fe_2O_3 crystal is held between a brass spring and a 5x1.5 mm FFKM - FFPM – 75 O-ring. The inner core is screwed inside the PEEK body to make a contact with the crystal. The whole holder is screwed inside a commercially available RDE shaft from Pine Research Instrumentation.

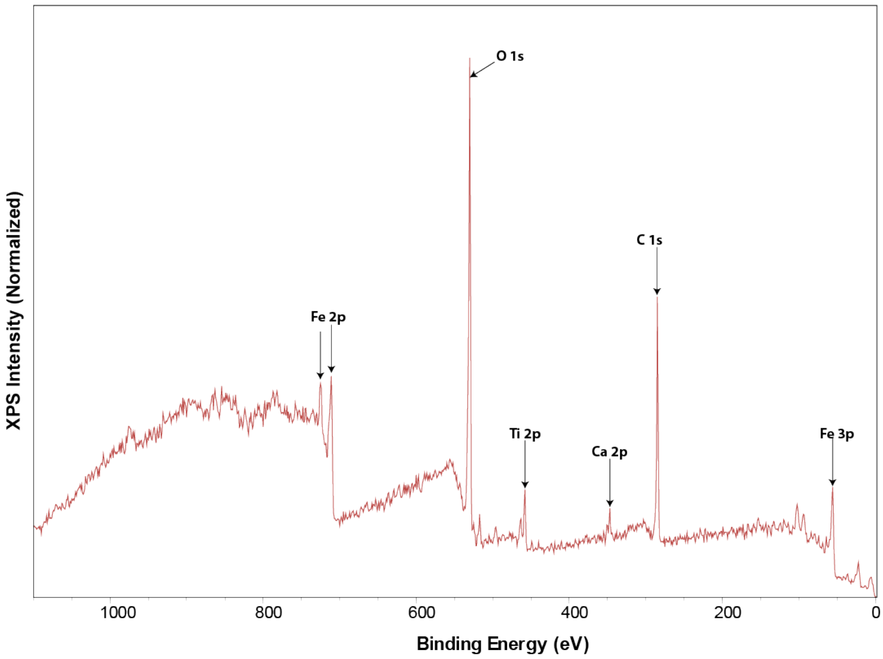

Figure S2: XPS survey of pristine Fe_2O_3(0001) single crystal showing dominant oxygen and iron peaks with some naturally occurring titanium and calcium.

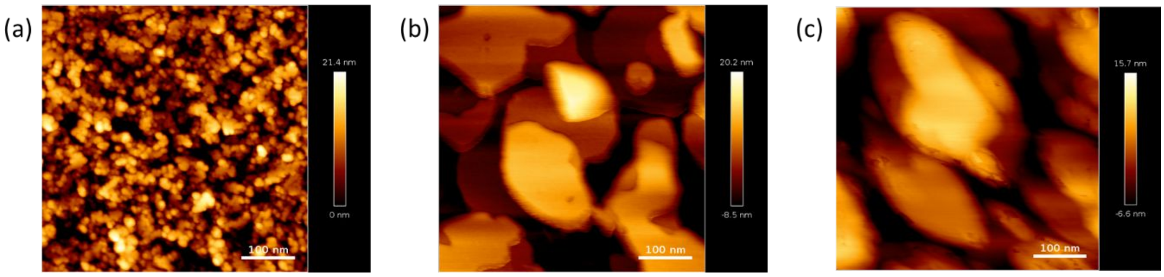

Figure S3: 1000x1000 nm AFM images taken in air with 512x512 resolution. (a) After roughening the surface by cycling between 0.1 V and 1.5 V. Subsequently, the surface was annealed (a) once and (b) twice at 1100 °C in air for 18 h. Even with annealing, the pristine surface before electrochemical roughening was not retrieved.

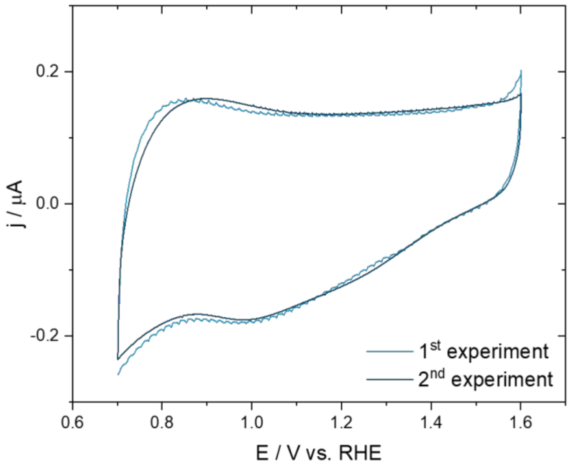

Figure S4: Comparison of the CV reproducibility with the same Fe_2O_3(0001) crystal 1 month apart. Electrolyte was 1 M NaOH. Between these two experiments, multiple scans and electrolytes were measured. Scan rate is 50 mV s^{-1}. The crystal was not annealed between these experiments. The largest reproducibility differences are observed around the Fe redox peaks <0.8V and in the OER region >1.55 V.

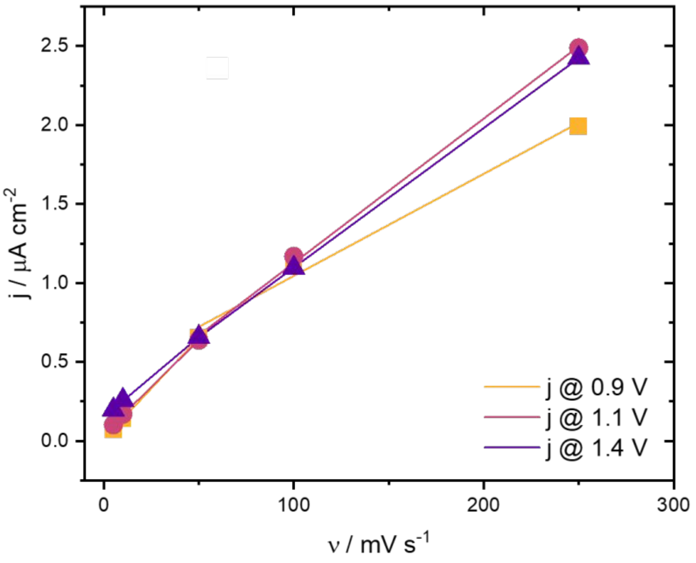

Figure S5: Scan rate dependent current at 0.9, 1.1, 1.4 V in 0.1 M NaOH fitted with two different slopes from 5 – 50 and 50 – 250 mV s^{-1} respectively.

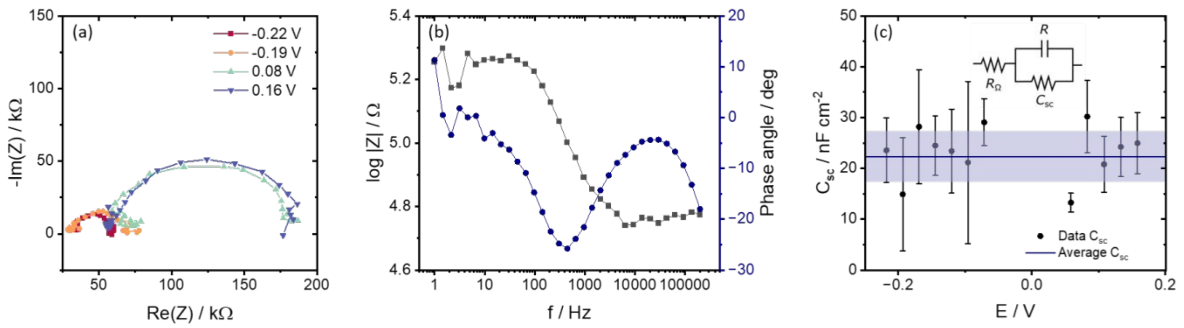

Figure S6: Bulk electrode impedance of a 2 mm thick Fe_2O_3(0001) crystal without any electrolyte present which was pressed between two gold electrodes. A different crystal than the one used in the main text was used because this measurement might have scratched the surface. Both crystals were obtained from the same supplier with the

same dimensions and the surfaces were both annealed as according to described in the experimental. (a) Nyquist plot of a $Fe_2O_3(0001)$ crystal under mild polarization measured between 200 kHz – 1 Hz and (b) representative Bode plot showing the existence of 1 semi-circle between 50 kHz – 10 Hz with a characteristic frequency of 500 Hz. This data was fitted using a simple Randles circuit (see inlay) showing (c) the potential independent C_{sc} of around 23 ± 5 nF cm^{-2}.

Table S1: Equivalent circuits fsor the hematite-electrolyte interface proposed in literature ranging from simple to more complicated models.

Model	Validation	References
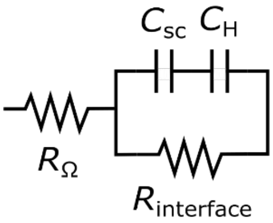	For highly concentrated electrolytes, the diffuse layer and the bulk resistance are neglected.	Hankin et al.[1]
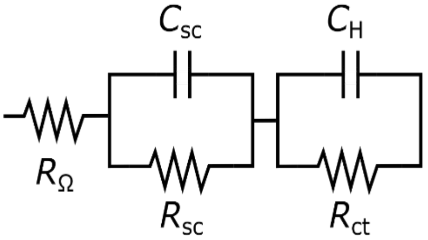	Simplified for electrodes in the dark including the bulk semiconductor resistance R_{sc}.	Lopes et al.[2]<br>Wielant et al.[3]
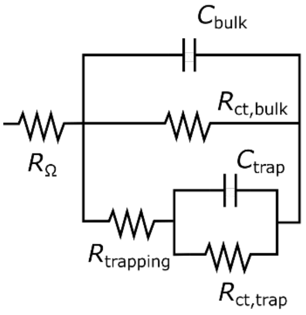	Electrodes under illumination highlighting the surface states and recombination centres for holes generated by light. Does not contain an electrolyte contribution.	Klahr et al.[4]
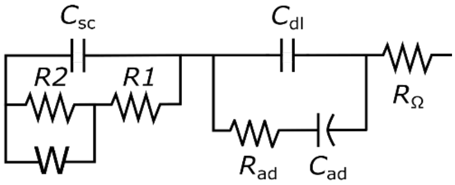	Expanded model with bulk resistance and charge diffusion as well as separate elements for the diffuse and compact layers of the double-layer.	Shimizu et al.[5]
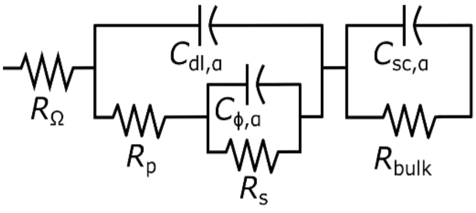	Expanded model applied to oxygen evolution reaction where adsorption of OER intermediates is included.	Lyons et al.[6]<br>Chakthranont et al.[7]

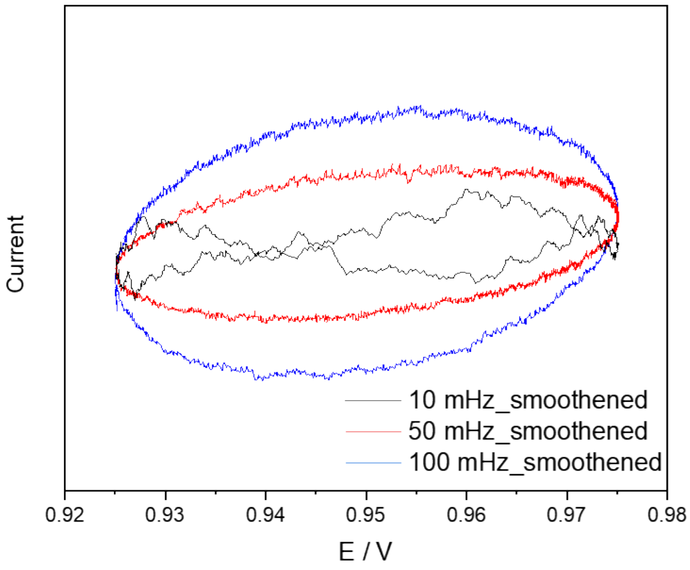

Figure S7: Lissajous plot taken in 10 mM NaOH with an amplitude of 50 mV at 10, 50 and 100 mHz showing that down to 10 mHz, the measured ac current is stable signifying that there is no drift in current between start and end of the oscillation.

1. Electrical equivalent circuit for capacitive and OER region

This section functions to explain and validate the usage of the electrical equivalent circuit used in the main text. First, a general explanation for the total impedance of the interface is given and then an analysis of the measured data is given to validate the usage of the electrical equivalent circuits.

In general, the total impedance response consists of a series of impedances that correspond to each element of the total system. The total impedance Z_{tot} consists of

$$Z_{\text{tot}} = Z_{\text{cell}} + Z_{\text{electrode}} + Z_{\text{interface}} + Z_{\text{counter}}. \tag{1}$$

In a three-electrode setup, current flows from the working electrode (WE) to the counter electrode (CE) and the potential is controlled relative to a reference electrode (RE) from which no current flows. Typically, the counter electrode surface area is sufficiently larger than the working electrode to not hinder the current flow or influence the impedance response. Therefore, the impedance of the interfacial charging and charge transfer at the counter electrode are not considered in the total impedance. Moreover, resistances, capacitances and inductances from the cables and potentiostat of the setup are typically negligible as they occur at very high frequencies.

As already established with Figure S6 and in the main text, the high frequency response of the bulk electrode can be modeled with a parallel RC circuit where the capacitance must be modeled with a constant phase element (CPE). Instead of a perfect capacitor with a phase angle of -90° where

$$Z_{\text{capacitor}} = -\frac{1}{j\omega C}, \tag{2}$$

j is $\sqrt{-1}$. α is introduced to account for the different phase angle, and Q replaces C as a pre-exponential factor with units F $s^{\alpha-1}$ or S s^{α} where $0 < \alpha < 1.0$. For a CPE,

$$Z_{\text{CPE}} = -\frac{1}{(j\omega)^{\alpha} Q}. \tag{3}$$

For a parallel R-CPE circuit, it is possible to determine the effective capacitance from the time constant τ where

$$\tau^{\alpha} = RQ = (RC)^{\alpha} \quad (5)$$

and R is the resistance parallel to the CPE associated with the space charge capacitance C. In turn, eq. 4 is used to account for the CPE behaviour for all the reported C_{sc} values in this paper.

$$C_{\mathrm{eff}} = \frac{(RQ)^{\frac{1}{\alpha}}}{R} \quad (5)$$

Therefore, the impedance of the electrode bulk is

$$Z_{\mathrm{electrode}} = \frac{R_{\mathrm{bulk}}}{1+R_{\mathrm{bulk}}*(j\omega)^{\alpha_{\mathrm{sc}}}*Q_{\mathrm{sc}}}. \quad (6)$$

Lastly, $Z_{\mathrm{interface}}$ considers the interfacial charging pathways originating from the electrode-electrolyte interface. The impedance response in Nyquist and Bode plots can be found in the main text Figure 4a and b. A zoom-in of the high frequency region in Figure S8a and b shows the similar high frequency impedance response as in Figure S6 which can be associated with the electrode itself. There is however some deviation around 10 kHz in the Bode plot between the experimental data and the fitted data which might arise from the limitations of the electrochemical cell with a high impedance or from the cell cables.[8–11] Nevertheless, the high frequency intercept with the Re(Z), which is the sum of solution resistance and contact resistance, is in the orders of a few tens of ohms which is expected for a solution of 0.1 M NaOH.

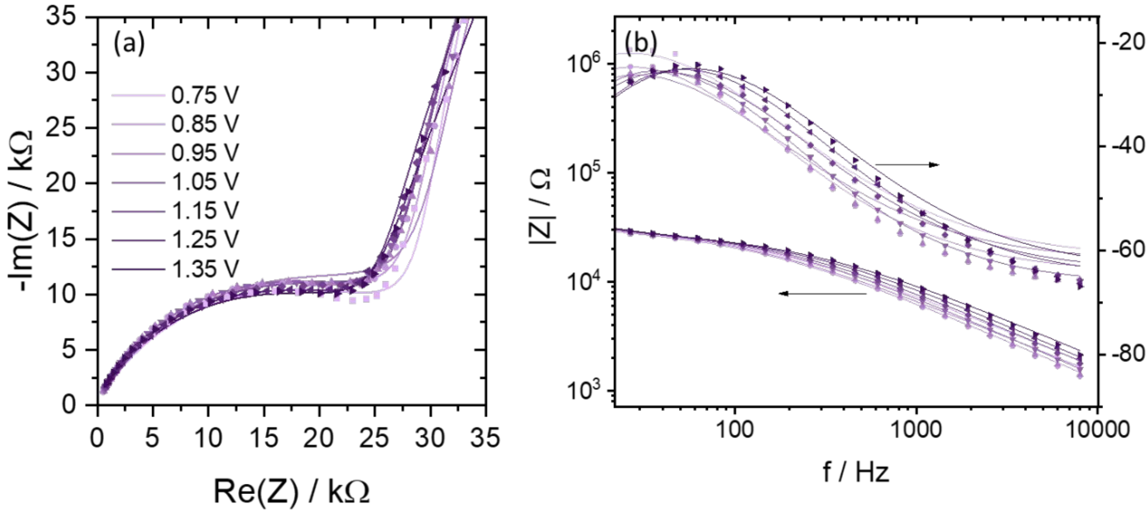

Figure S8: (a) Zoom-in of the Nyquist impedance plot in 0.1 M NaOH from main text Figure 4a showing the impedance of the bulk electrode (points) and the fitted model (line). (b) Zoom-in of the Bode impedance plot in 0.1 M NaOH from main text Figure 4a showing the impedance data (points) of the bulk electrode and the fitted model (line).

Unlike Figure S6, the impedance response in Figure S8 deviates from a semicircle at frequencies below ~50 Hz. From ~50 Hz to just below 1 Hz, a second semicircle can be seen in the Nyquist plot and half a peak from -20° to -60° can be seen in the Bode plot in main Figure 4b. These features are associated with a parallel RC circuit such as for the bulk electrode, but the capacitance and resistance values are distinctly different than those of the space charge capacitance and the bulk resistance respectively.[10] If they were similar, the impedance would have been similar, but the mid-frequency region is clearly distinct from the high frequency impedance. However, unlike the bulk, the phase angle remains relatively constant below -60° below 1 Hz which indicates a dominant capacitive behaviour. Therefore, there can be a constant phase element or capacitance in the EEC that is not parallel to any resistance but rather in series.

Our circuits were kept as simple as possible to avoid overfitting. The simplest circuit to be able to model the observed data is the one given in Figure 4c. Whereas the $R_\Omega - C_{dl}/(R_{ct} - C_{ad})$ and R_Ω - $C_{dl}/R_{ct} - C_{ad}$ circuits could produce the same impedance response with the right parameters, R_Ω - $C_{dl}/R_{ct} - C_{ad}$ assumes that the electrical pathway $R_\Omega - C_{dl} - C_{ad}$ is valid. This implies that an adsorbate can adsorb without the transfer of charge through the interface. To separate the double layer capacitance from an adsorption pathway with interfacial charge transfer, we used $R_\Omega - C_{dl}/(R_{ct} - C_{ad})$ for the fitting of the mid to low frequency regime.

A Warburg element, W, for which the impedance is derived as:

$$Z = \frac{A_W}{\sqrt{2\pi f}}(1 - j) \tag{7}$$

could replace or be added in series to the C_{ad} in case the low frequency feature would be a diffusion limited or intercalation related process. This case for different fitting parameters is shown in Figure S9 where R_Ω-C_{sc}/R_{bulk}-$C_{ad}/(R_{ct}$-W-$C_{ad})$ is shown in Figure S9a and R_Ω-C_{sc}/R_{bulk}-$C_{ad}/(R_{ct}$-$W)$ is shown in Figure S9b. Only Figure S9a for parameter set 1-3 closely resembles our measured data in the limit where the Warburg element is almost negligible and therefore it is not required for a sufficient fit. As can be seen from Figure S9b, no values for A_w are satisfactory to describe the kind of mid-to-low frequency behaviour observed in this system and therefore no Warburg elements are included in our circuit.

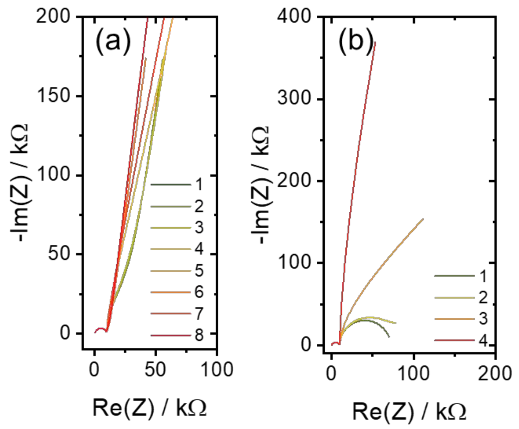

(c)

R_{ct}-W	C_{dl} / µF	R_{ct} / kΩ	C_{ad} / µF	C_{sc} / nF	R_{bulk} / kΩ	A_W / Ω $s^{-1/2}$
1	6	60	-	10	100	100
2	6	60	-	10	100	1000
3	6	60	-	10	100	10000
4	6	60	-	10	100	100000
**R_{ct}-W-CPE**	**C_{dl} / µF**	**R_{ct} / kΩ**	**C_{ad} / µF**	**C_{sc} / nF**	**R_{bulk} / kΩ**	**A_W / Ω $s^{-1/2}$**
1	6	60	8	10	100	10
2	6	60	8	10	100	1000
3	6	60	8	10	100	10000
4	6	60	8	10	100	100000
5	6	10	8	10	100	100
6	6	10	8	10	100	10000
7	6	10	8	10	100	100000

Figure S9: (a) Simulated EIS data using the R_Ω-C_{sc}/R_{bulk}-$C_{ad}/(R_{ct}$-W-$CPE_{ad})$ circuit with different fitting parameters in c. (b) Simulated EIS data using the R_Ω-C_{sc}/R_{bulk}-$C_{ad}/(R_{ct}$-$W)$ circuit with different fitting parameters from c. (c) Fitting parameters for different simulated conditions.

More parallel elements for phenomena such as surface states, hole diffusion, different adsorption processes, or additional parallel charge transfer could be added but these would unnecessarily complicate the circuit.[4,12] Therefore, the EEC R_Ω-C_{sc}/R_{bulk}-$C_{ad}/(R_{ct}$-$CPE_{ad})$ was chosen, which extends to the one proposed by Lyons et al.[6] and Chakthranont et al.[7] in case of OER as demonstrated in Figure S11. The full impedance expression is then

$$Z_{total} = R_\Omega + \frac{R_{bulk}}{1 + R_{bulk}(j\omega)^{\alpha_{sc}} Q_{sc}} + \frac{1}{(j\omega)^{\alpha_{ad}} Q_{ad} + \frac{j\omega C_{dl}}{j\omega C_{dl} R_{ct} + 1}}. \tag{8}$$

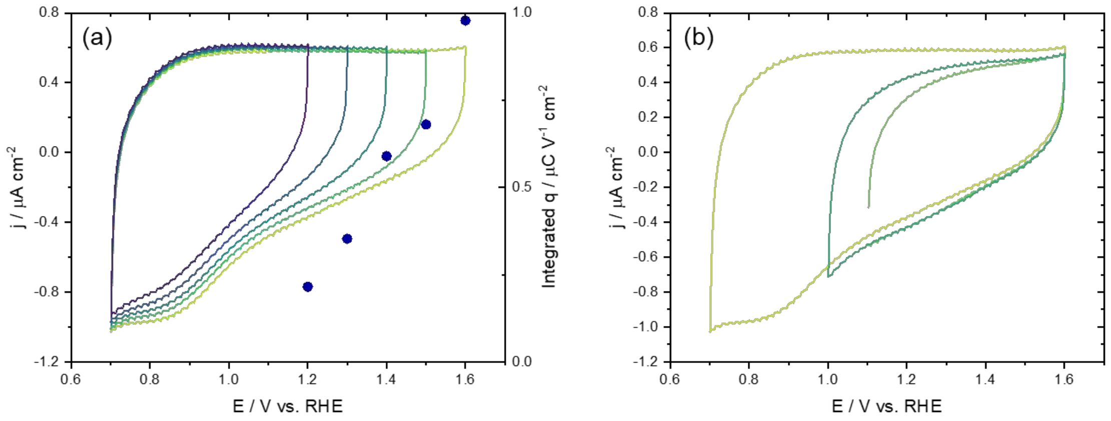

Figure S10: (a -b) Effects of the scan range on the obtained current at pH 11 in 1 M $NaClO_4$ background current and integrated charge density (blue dots) from 0.7 to the upper vertex potential normalized by the total potential region showing the CV is most reversible from 0.7 – 1.2 V and least reversible from 1.1 – 1.6 V.

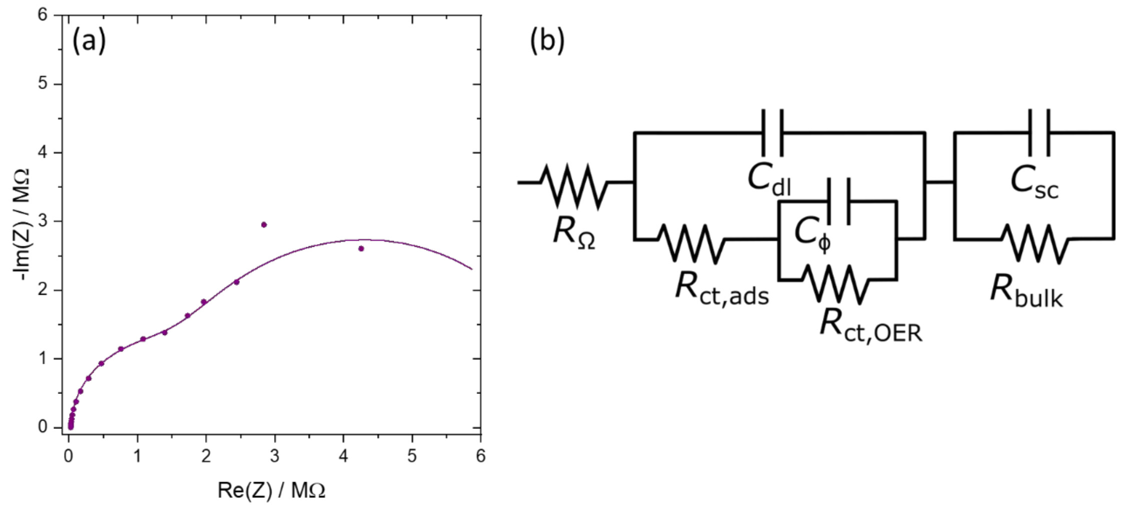

Figure S11: (a) Nyquist impedance plot under OER conditions in 0.1 M NaOH. (b) Suggested EEC for a metal oxide under OER conditions where C_{Φ} and $R_{ct,OER}$ are used to model the charge involved with the adsorption of OER intermediates.[6]

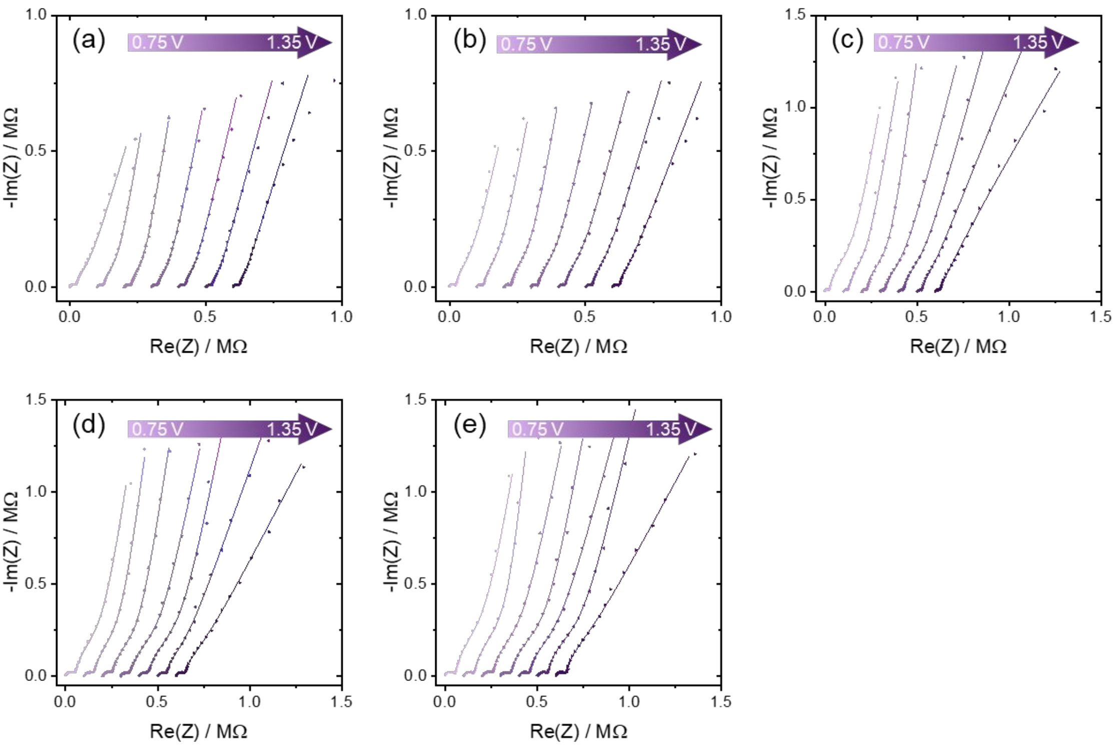

*Figure S12: Experimental EIS data and their fitted EIS spectra to the EECs in Figure 4a and b from 25 kHz – 50 mHz at potentials ranging from 0.75 – 1.25 V vs. RHE (– for model), (symbols for experimental data)in (a) 1 M NaOH, (b) 0.3 M NaOH, (c) 0.1 M NaOH, (d) 0.03 M NaOH and (e) 0.01 M NaOH. Every subsequent dataset per potential is offset by 100 k*Ω *on the Re(Z) axis with respect to the previous potential.*

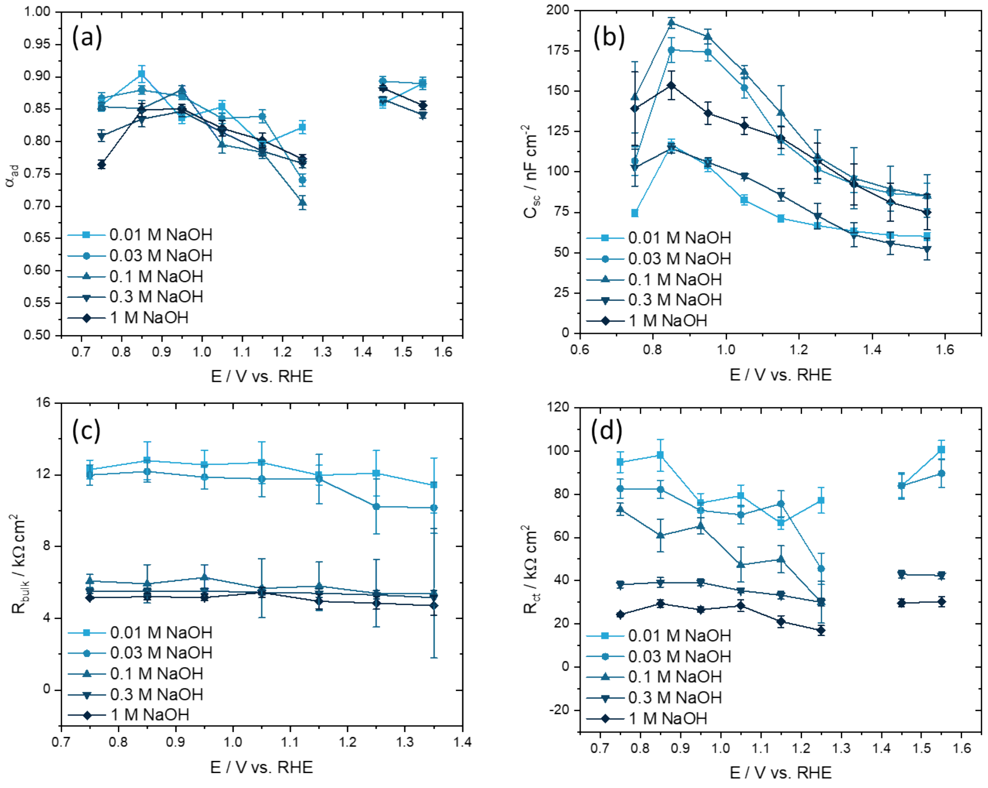

Figure S13: (a) CPE exponent for C_{ad}, (b) space charge capacitance, (c) bulk resistance and (d) charge transfer resistance as obtained from fitting the EIS spectra to the EEC circuit in main figure 4c-d, in different NaOH concentrations; frequencies between 25 kHz – 100 Hz. (d)

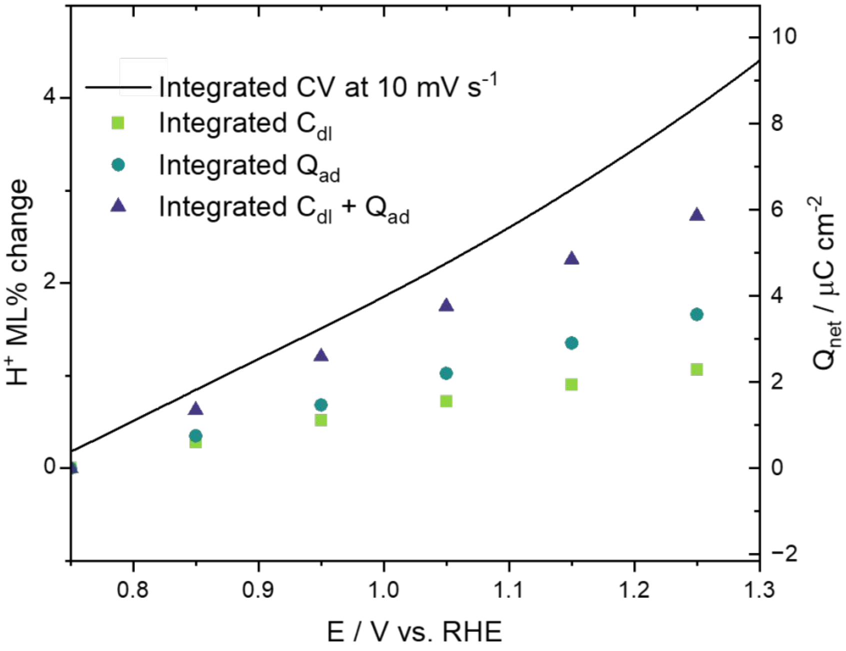

Figure S14: Decrease in H^{+}-charge from 0.75 V to 1.25 V in 0.1 M NaOH by using the integrated CV and EIS fitting parameters.

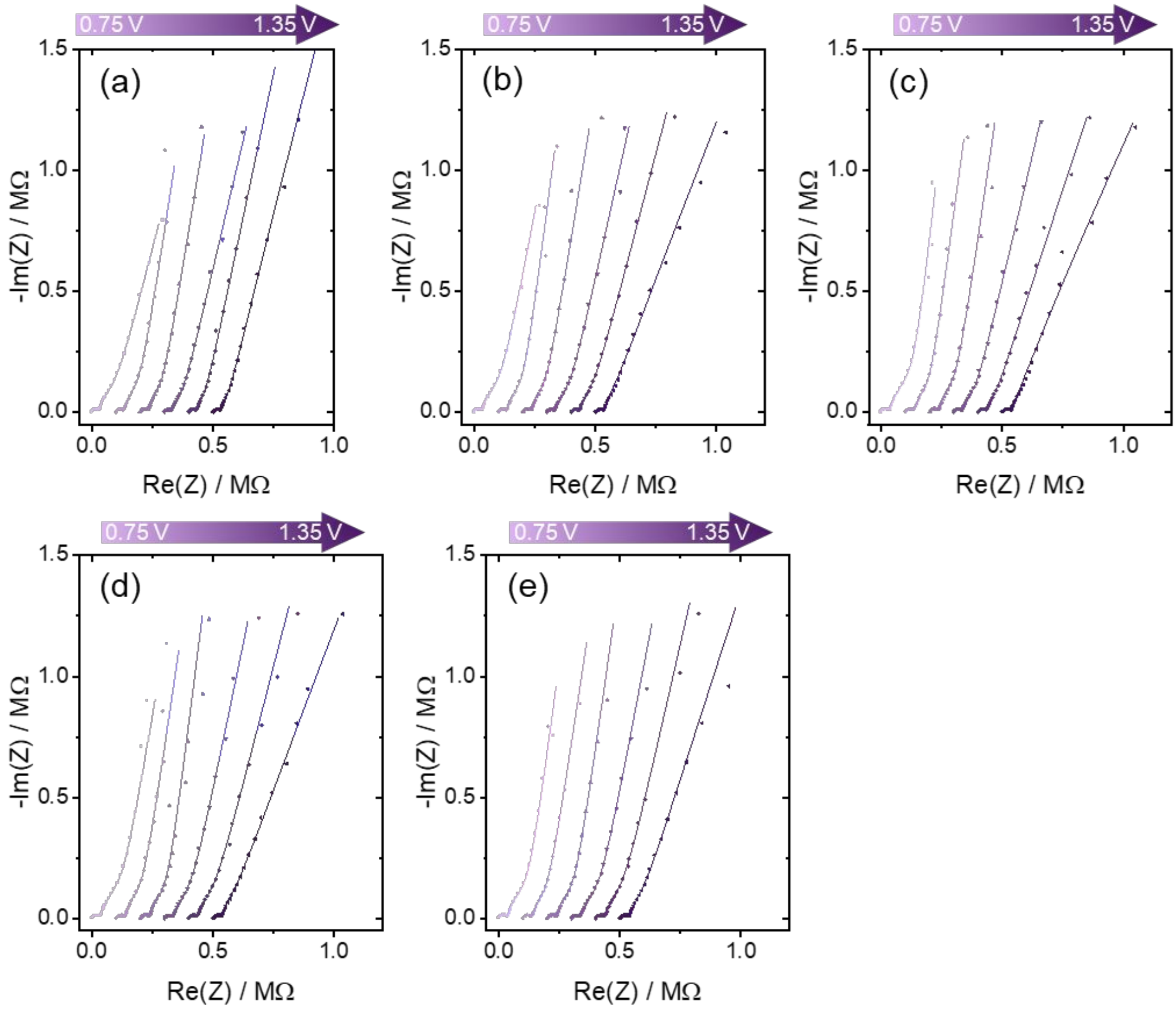

Figure S15: Experimental EIS data and their fitted EIS spectra to the EECs in Figure 4a and b from 25 kHz – 50 mHz at potentials ranging from 0.75 – 1.25 V vs. RHE (– for model), (symbols for experimental data) in NaOH plus the appropriate $NaClO_4$ concentration to have $[Na^+]$ = 1.0 M, for (a) pH 14.0 (≈13.7), (b) pH 13.5, (c) pH 13.0, (d) pH 12.5 and (e) pH 12. Every subsequent dataset per potential is offset by 100 kΩ on the Re(Z) axis with respect to the previous potential.

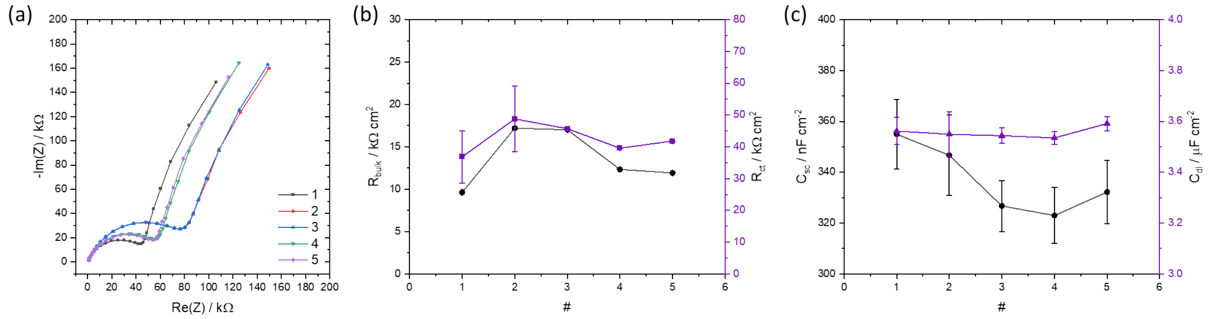

Figure S16: Impedance response reproducibility at 1.1 V in 0.01 M NaOH for 5 different meniscus setups showing the variation in (a) raw impedance response at a fixed potential and (b-c) the variation in fitting parameters R_{bulk}, C_{dl} and C_{sc} by using the EEC in Figure 4a where the error bar denotes the accuracy of the fit.

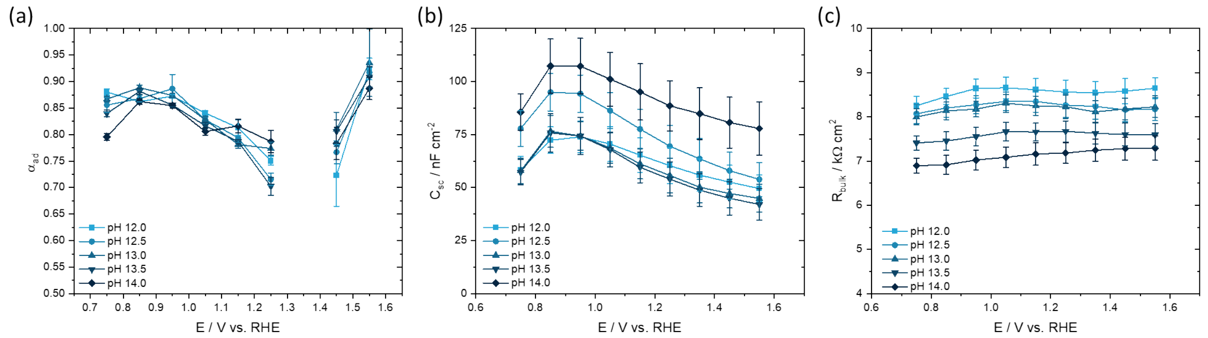

Figure S17: (a) CPE exponent for C_{ad}, (b-c) space charge capacitance and bulk resistance as obtained from fitting the EIS spectra to the EEC circuit in Figure 4 a and b, in NaOH plus the appropriate $NaClO_4$ concentration to have $[Na^+]$ = 1.0 M, at different pH. Frequency range between 25 kHz – 100 Hz.

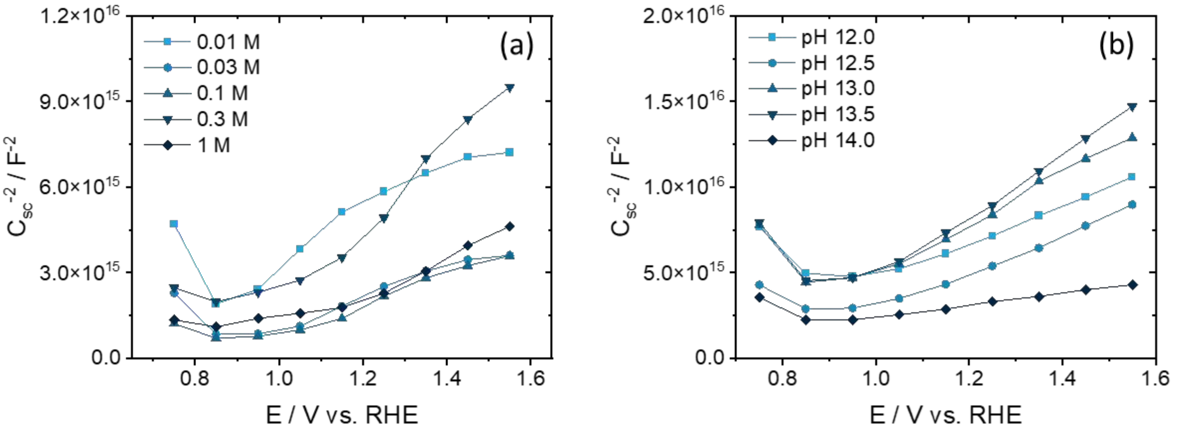

Figure S18: Mott-Schottky plots as obtained from fitting the EIS spectra to the EEC circuit in Figure 4 a and b, in (a) pure 0.01 – 1 M NaOH and (b) at pH 12 – 14 (NaOH + $NaClO_4$), $[Na^+]$ = 1 M. Frequency range between 25 kHz – 100 Hz.

2. Mott-Schottky analysis

For analysing the EIS data at high frequency, C_{sc} was measured as a function of [NaOH] and pH (see Figure S17b), and $1/C_{sc}^2$ was plotted against the potential in a Mott-Schottky (MS) plot (Figure S18).[13] While the linear region is quite limited, a linear relationship exists between $1/C_{sc}^2$ and E. Using the Mott-Schottky equation (eq. 2 in the main text) the charge carrier density, N_d, and the flat band potentials (intercepts) were calculated using ε = 32, and they are shown in Figure S19.[4] E_{FB}, N_d, were averaged over all electrolyte concentrations and they are tabulated in Table S2. Moreover, using N_d, the space-charge layer width, W, was calculated:

$$W = \sqrt{\frac{2\varepsilon\varepsilon_0(E-E_{FB})}{N_D e}} \tag{9}$$

and this is also reported in Table S2 for 0.3 V space-charge polarization (corresponding to applied potentials between 1.0 – 1.2 V vs. RHE depending on the pH).

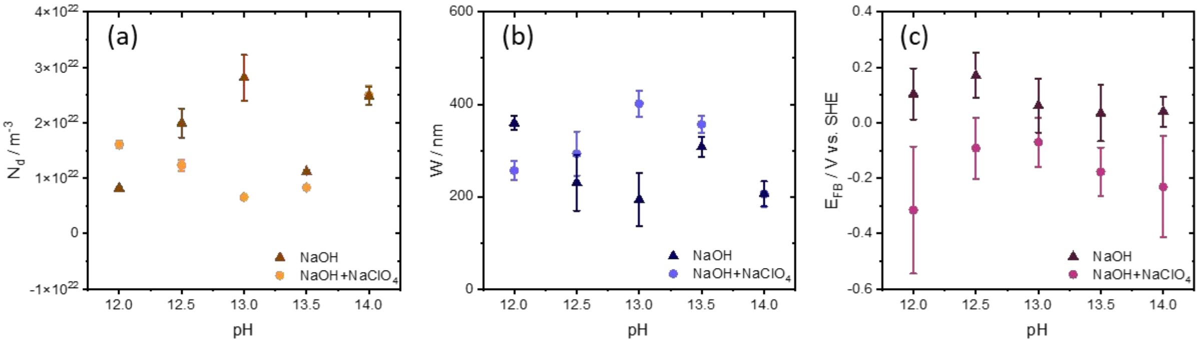

Figure S19: Mott-Schottky plot parameters obtained in between pH 12-14 with and without $[Na^+]$ = 1 M from the Mott-Schottky plots in Figure S18. The average of the values is given in Table S2. (a) Charge carrier density N_d, (b) space charge region width W and (c) flat band potential E_{FB} (intercept of Mott-Schottky plot).

Similar values for N_d and W were found around 1.6 ± 0.9 * 10^{22} m^{-3} and 281 ± 95 nm, respectively for both NaOH and NaOH + $NaClO_4$ electrolytes in Table S2. Reported values for N_d in literature are 10^{25} – 10^{26} m^{-3} and 10^{20} – 10^{25} m^{-3} for respectively doped and undoped hematite samples.[1,14–17] Our charge carrier density of ~10^{22} m^{-3} matches more closely the lower charge carrier density end of the undoped samples, which suggests an undoped sample in which electrons in the conduction band are the main conductors in the bulk.

Table S2: Flat band potentials and charge carrier densities for Fe_2O_3 in NaOH and $NaClO_4$ containing electrolytes obtained from a Mott-Schottky analysis (Figure S18).

Electrolyte	E_{FB} V vs. SHE	N_d[a] / m^{-3}	W / nm @ $E - E_{FB}$ = 0.3 V
0.01 – 1 M NaOH	0.08 ± 0.05	1.8 ± 0.8 * 10^{22}	259 ± 64
pH 12 – 14 (NaOH + $NaClO_4$), $[Na^+]$ = 1 M	-0.17 ± 0.09	1.4 ± 0.7 * 10^{22}	303 ± 70

[a] *Calculated using the Mott-Schottky equation for pH 12 – 14 and ε = 32 (eq. 2 in the main text).*[4]

Overall, the intercepts of the Mott-Schottky analysis were found to be relatively independent of electrolyte composition and slightly negative of 0 V vs. SHE (Table S2 & Figure S19). However, a discrepancy emerged during data reproduction for 1 M NaOH: the flat band potentials were measured as 0.04 ± 0.05 V and -0.23 ± 0.09 V vs. SHE respectively while N_d and W were identical for both measurements. Similarly, measurements in $NaClO_4$-containing electrolytes showed a ~0.2 V shift to more negative flat-band potentials compared to pure NaOH, while other MS parameters remained unchanged. This shift likely stems from variations in the working electrode assembly and resulting high frequency behaviour. Such differences in the high frequency behaviour were also observed when refreshing the contact between the working electrode and the electrolyte multiple times for the same electrolyte (Figure S16), where the standard deviation was > 20%, < 1%, ~10% and ~3% of the average measured value for R_{bulk}, C_{dl}, R_{ct} and C_{sc} respectively.

It also must be noted that the crystal was only removed from the electrode holder when the whole concentration series experiment had finished. Thus, E_{FB} was constant within experimental deviation whenever the working electrode remained assembled However, the electrode holder was cleaned and reassembled between experiments. Therefore, reassembly could have led to a constant shift in E_{FB} outside of the experimentally observed deviation, while other parameters, such as the slope of the Mott-Schottky plot, remained constant within experimental deviation. It is possible that small contact differences in the assembly of the working electrode could have led to a constant shift in the intercept between the two experiments. Alternatively, changes in μ_1-OH coverage due to aging might have contributed to the observed shift.[18] Therefore, there is no strong evidence to suggest that the electrolyte composition significantly influences the semiconducting properties of single crystalline α-Fe_2O_3.

Despite the small shift in the Mott-Schottky plot intercept, outside of experimental deviation, when introducing more Na^+ in the electrolyte, E_{FB} consistently remained slightly negative of 0 V vs. SHE (Table S2) across all electrolytes. Compared to literature, our flat band values are 300 – 500 mV more positive.[1] However, as highlighted in other studies, E_{FB} cannot be measured directly, and variances in methodologies can lead to different values and interpretations.[1,19] For instance, fitting a simple EEC model can yield an entirely different E_{FB} than by using photo-electrochemical methods. This inherent methodological variability introduces unavoidable uncertainties in determining E_{FB}.

Interestingly, both our flat-band potential values and those from the literature do not lie between the valence and conduction band edges, as predicted by the MS theory.[19] Instead, the 'apparent' flat band potential lies more negative than both the conduction and valence band edges at the flat band condition which are respectively $E_{C, FB} = 0.88 \pm 0.29 - 0.059*pH = 0.113 \pm 0.29$ V vs. SHE at pH 13, and $E_{V, FB} = 2.93 \pm 0.44 - 0.059*pH = 2.163 \pm 0.44$ V vs. SHE as calculated by Hankin et al.[1] However, as Hankin et al. stated in their paper, the band position calculations neglected the adsorption of species other than protons and hydroxide ions.

Since impurities have been ruled out, the unusually low E_{FB} could more likely be explained by surface-state-mediated band bending rather than the band bending behaviour of an intrinsic semiconductor. Surface states may arise from the crystal structure termination, oxygen defects or interactions with the electrolyte.[20] Undercoordinated Fe/O sites, protonated μ_2-O(H) groups, or more complex species such as iron-peroxide species formed under illumination could introduce energy states within or near the bandgap.[4,12,21] Although photoinduced surface states are unlikely in our experiments due to the absence of a strong light source, the observed pseudocapacitive cyclic voltammetry response and unusual E_{FB} suggests the presence of surface states, potentially in the form of surface trapped holes originating from the O-terminated surface with adsorbed H^+ charges.[1,4,22]

These surface states, with energy levels within the bandgap, alter the electronic structure of the electrode-electrolyte interface by localizing charges from the space charge region at the surface.[23,24] Furthermore, because the interfacial cation concentration in a Helmholtz layer (10^{25}-10^{26} m^{-3}) and the surface coverage of H^+ are larger than the bulk charge carrier density (~10^{22} m^{-3}), the Fermi level is pinned by the surface states and independent of the bulk concentration.[23,25] Therefore, the charging of the space charge region becomes less relevant to the total interfacial charging and the interface begins to resemble the metal-electrolyte interface.[24] As a result, the potential difference across the interface will not be dominantly located in the space charge region anymore, but more at the surface. Consequently, the potential drop at the interface will be Helmholtz-like,[24] which also agrees with our results.

References

1. Hankin, A., Alexander, J. C. & Kelsall, G. H. Constraints to the flat band potential of hematite photo-electrodes. *Physical Chemistry Chemical Physics* **16**, 16176–16186 (2014).
2. Lopes, T., Andrade, L., Le Formal, F., Gratzel, M., Sivula, K. & Mendes, A. Hematite photoelectrodes for water splitting: evaluation of the role of film thickness by impedance spectroscopy. *Physical Chemistry Chemical Physics* **16**, 16515–16523 (2014).
3. Wielant, J., Goossens, V., Hausbrand, R. & Terryn, H. Electronic properties of thermally formed thin iron oxide films. *Electrochimica Acta* **52**, 7617–7625 (2007).
4. Klahr, B., Gimenez, S., Fabregat-Santiago, F., Hamann, T. & Bisquert, J. Water Oxidation at Hematite Photoelectrodes: The Role of Surface States. *Journal of the American Chemical Society* **134**, 4294–4302 (2012).

5. Shimizu, K. & Boily, J.-F. Electrochemical Properties and Relaxation Times of the Hematite/Water Interface. *Langmuir* **30**, 9591–9598 (2014).
6. Lyons, M. E. G. & Brandon, M. P. The significance of electrochemical impedance spectra recorded during active oxygen evolution for oxide covered Ni, Co and Fe electrodes in alkaline solution. *Journal of Electroanalytical Chemistry* **631**, 62–70 (2009).
7. Chakthranont, P., Kibsgaard, J., Gallo, A., Park, J., Mitani, M., Sokaras, D., Kroll, T., Sinclair, R., Mogensen, M. B. & Jaramillo, T. F. Effects of Gold Substrates on the Intrinsic and Extrinsic Activity of High-Loading Nickel-Based Oxyhydroxide Oxygen Evolution Catalysts. *ACS Catalysis* **7**, 5399–5409 (2017).
8. Fröhlich, N. L., Eggebeen, J. J. J. & Koper, M. T. M. Measurement of the double-layer capacitance of Pt(111) in acidic conditions near the potential of zero charge. *Electrochimica Acta* **494**, 144456 (2024).
9. Tran, A.-T., Huet, F., Ngo, K. & Rousseau, P. Artefacts in electrochemical impedance measurement in electrolytic solutions due to the reference electrode. *Electrochimica Acta* **56**, 8034–8039 (2011).
10. Wang, S., Zhang, J., Gharbi, O., Vivier, V., Gao, M. & Orazem, M. E. Electrochemical impedance spectroscopy. *Nature Reviews Methods Primers* **1**, 41 (2021).
11. Battistel, A., Fan, M., Stojadinović, J. & La Mantia, F. Analysis and mitigation of the artefacts in electrochemical impedance spectroscopy due to three-electrode geometry. *Electrochimica Acta* **135**, 133–138 (2014).
12. Li, J., Wan, W., Triana, C. A., Chen, H., Zhao, Y., Mavrokefalos, C. K. & Patzke, G. R. Reaction kinetics and interplay of two different surface states on hematite photoanodes for water oxidation. *Nature Communications* **12**, 255 (2021).
13. Gelderman, K., Lee, L. & Donne, S. W. Flat-Band Potential of a Semiconductor: Using the Mott–Schottky Equation. *Journal of Chemical Education* **84**, 685 (2007).
14. Chatman, S., Zarzycki, P. & Rosso, K. M. Surface potentials of (001), (012), (113) hematite (α-Fe2O3) crystal faces in aqueous solution. *Physical Chemistry Chemical Physics* **15**, 13911–13921 (2013).

15. Chatman, S., Pearce, C. I. & Rosso, K. M. Charge Transport at Ti-Doped Hematite (001)/Aqueous Interfaces. *Chemistry of Materials* **27**, 1665–1673 (2015).

16. Kumari, S., Tripathi, C., Singh, A. P., Chauhan, D., Shrivastav, R., Dass, S. & Satsangi, V. R. Characterization of Zn-doped hematite thin films for photoelectrochemical splitting of water. *Current Science* **91**, 1062–1064 (2006).

17. Cesar, I., Sivula, K., Kay, A., Zboril, R. & Grätzel, M. Influence of Feature Size, Film Thickness, and Silicon Doping on the Performance of Nanostructured Hematite Photoanodes for Solar Water Splitting. *The Journal of Physical Chemistry C* **113**, 772–782 (2009).

18. Lützenkirchen, J., Heberling, F., Supljika, F., Preocanin, T., Kallay, N., Johann, F., Weisser, L. & J. Eng, P. Structure–charge relationship – the case of hematite (001). *Faraday Discussions* **180**, 55–79 (2015).

19. Hankin, A., Bedoya-Lora, F. E., Alexander, J. C., Regoutz, A. & Kelsall, G. H. Flat band potential determination: avoiding the pitfalls. *Journal of Materials Chemistry A* **7**, 26162–26176 (2019).

20. Warschkow, O., Ellis, D. E., Hwang, J., Mansourian-Hadavi, N. & Mason, T. O. Defects and Charge Transport near the Hematite (0001) Surface: An Atomistic Study of Oxygen Vacancies. *Journal of the American Ceramic Society* **85**, 213–220 (2002).

21. Memming, R. Charge Transfer Processes at the Semiconductor–Liquid Interface. in *Semiconductor Electrochemistry* 169–266 (2015). doi:https://doi.org/10.1002/9783527688685.ch7.

22. Zhang, Y., Zhang, H., Ji, H., Ma, W., Chen, C. & Zhao, J. Pivotal Role and Regulation of Proton Transfer in Water Oxidation on Hematite Photoanodes. *J. Am. Chem. Soc.* **138**, 2705–2711 (2016).

23. Zhang, Z. & Yates, J. T. Jr. Band Bending in Semiconductors: Chemical and Physical Consequences at Surfaces and Interfaces. *Chemical Reviews* **112**, 5520–5551 (2012).

24. Bockris, J. O., Reddy, A. K. N. & Gamboa-Aldeco, M. E. *Modern Electrochemistry 2B: Electrodics in Chemistry, Engineering, Biology and Environmental Science*. (Springer Science & Business Media, 1998).

25. Memming, R. Principles of Semiconductor Physics. in *Semiconductor Electrochemistry* 1–22 (2015).
